# G_q_ signaling in **α** cells is critical for maintaining euglycemia

**DOI:** 10.1172/jci.insight.152852

**Published:** 2021-12-22

**Authors:** Liu Liu, Diptadip Dattaroy, Katherine F. Simpson, Luiz F. Barella, Yinghong Cui, Yan Xiong, Jian Jin, Gabriele M. König, Evi Kostenis, Jefferey C. Roman, Klaus H. Kaestner, Nicolai M. Doliba, Jürgen Wess

**Affiliations:** 1 Molecular Signaling Section, Laboratory of Bioorganic Chemistry, National Institute of Diabetes and Digestive and Kidney Diseases, NIH, Bethesda, Maryland, USA.; 2 Mount Sinai Center for Therapeutics Discovery, Departments of Pharmacological Sciences and Oncological Sciences and Neuroscience, Tisch Cancer Institute, Icahn School of Medicine at Mount Sinai, New York, New York, USA.; 3 Institute of Pharmaceutical Biology, University of Bonn, Bonn, Germany.; 4 Institute for Diabetes, Obesity and Metabolism, Perelman School of Medicine, University of Pennsylvania, Philadelphia, Pennsylvania, USA.

**Keywords:** Metabolism, G protein–coupled receptors

## Abstract

Glucagon, a hormone released from pancreatic α cells, plays a key role in maintaining euglycemia. New insights into the signaling pathways that control glucagon secretion may stimulate the development of novel therapeutic agents. In this study, we investigated the potential regulation of α cell function by G proteins of the G_q_ family. The use of a chemogenetic strategy allowed us to selectively activate G_q_ signaling in mouse α cells in vitro and in vivo. Acute stimulation of α cell G_q_ signaling led to elevated plasma glucagon levels, accompanied by increased insulin release and improved glucose tolerance. Moreover, chronic activation of this pathway greatly improved glucose tolerance in obese mice. We also identified an endogenous G_q_-coupled receptor (vasopressin 1b receptor; V1bR) that was enriched in mouse and human α cells. Agonist-induced activation of the V1bR strongly stimulated glucagon release in a G_q_-dependent fashion. In vivo studies indicated that V1bR-mediated glucagon release played a key role in the counterregulatory hyperglucagonemia under hypoglycemic and glucopenic conditions. These data indicate that α cell G_q_ signaling represents an important regulator of glucagon secretion, resulting in multiple beneficial metabolic effects. Thus, drugs that target α cell–enriched G_q_-coupled receptors may prove useful to restore euglycemia in various pathophysiological conditions.

## Graphical abstract

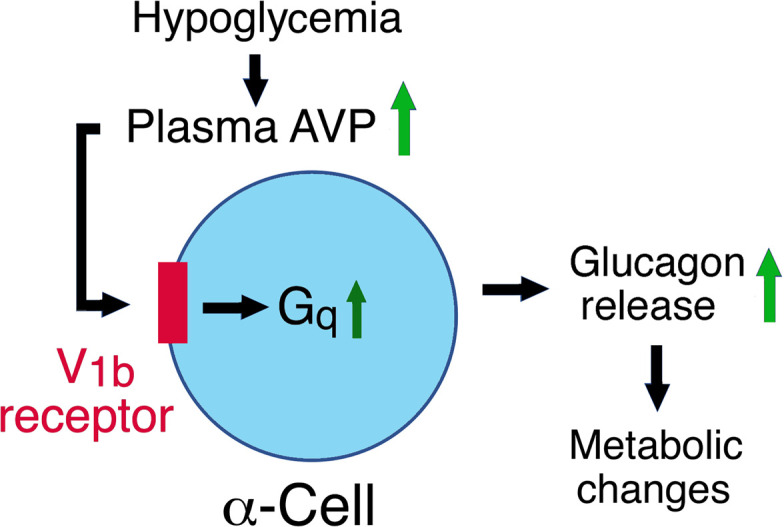

## Introduction

Glucagon is a metabolically important polypeptide hormone that is secreted from α cells of the pancreatic islets of Langerhans (1–5). During times of fasting, circulating glucagon increases blood glucose levels primarily by activating hepatic glucagon receptors, resulting in enhanced hepatic glucose output (1–5). Previous work has demonstrated that α cell dysfunction contributes to impaired glucose homeostasis in both type 1 diabetes and type 2 diabetes (T2D) (1, 3, 5, 6). A better understanding of α cell physiology and pathophysiology may lead to the development of novel drugs designed to improve α cell function for therapeutic purposes.

During the past few years, a series of important new findings have greatly stimulated research in the glucagon/α cell field. Several groups reported that glucagon released from α cells can promote the secretion of insulin from adjacent β cells in a paracrine fashion (7–10). Additional work suggested that this paracrine glucagon effect is mediated primarily by glucagon activation of β cell glucagon-like peptide 1 (GLP-1) receptors (7, 8). A more recent study clearly demonstrated that glucagon acts as an insulinotropic hormone in the fed state, enhancing rather than opposing the ability of insulin to maintain euglycemia (11). In line with these recent findings, agents that can simultaneously activate GLP-1 and glucagon receptors (so-called dual agonists) are currently undergoing clinical trials as potential novel drugs for the treatment of T2D and related metabolic disorders (12, 13).

The neurotransmitters, hormones, paracrine factors, and neuronal pathways that regulate β cell function have been explored in considerable detail. In contrast, much less is known about the factors, receptors, and signaling pathways and molecules that affect the function of pancreatic α cells (10). However, such knowledge may lead to novel approaches to modulate α cell function for therapeutic purposes.

Like virtually all other cell types, pancreatic α cells express a large number of G protein–coupled receptors (GPCRs) (14). GPCRs represent excellent drug targets, as indicated by the fact that about 35% of all FDA-approved drugs act on this class of receptors (15). Following the binding of extracellular ligands, GPCRs activate distinct functional classes of heterotrimeric G proteins (16, 17). Heterotrimeric G proteins are subdivided into 4 major subfamilies, G_s_, G_i/o_, G_q/11_, and G_12/13_ (16), primarily due to the signaling properties of the G protein α subunits. Previous studies have shown that activation of α cell G_i_ signaling strongly suppresses glucagon release (5, 7, 14), resulting in pronounced changes in glucose homeostasis. On the other hand, relatively little is known about the in vivo metabolic roles of α cell G_s_, G_q/11_, or G_12/13_ signaling.

The present study was designed to elucidate the in vivo metabolic changes caused by selective stimulation of α cell G_q_ signaling. Specifically, we employed a chemogenetic approach to generate a potentially new mouse model that expresses a G_q_-coupled designer GPCR (G_q_ DREADD; G_q_-coupled designer receptor exclusively activated by a designer drug; alternative names: hM3Dq or simply GqD) (18, 19) selectively in α cells (α-GqD mice). Treatment of α-GqD mice with clozapine-*N*-oxide (CNO) (18, 20) or deschloroclozapine (DCZ), a more potent and metabolically more stable CNO derivative (21), leads to the selective stimulation of the GqD designer receptor. When used in the proper dose or concentration range, CNO and DCZ are otherwise pharmacologically inert (18, 19, 21). Metabolic studies with α-GqD mice showed that activation of α cell G_q_ signaling led to a robust increase in glucagon and insulin secretion in vitro and in vivo. Importantly, we demonstrated that chronic activation of this pathway greatly improved glucose homeostasis in obese, glucose-intolerant mice.

Prompted by these findings, we identified an endogenous G_q_-coupled receptor, the vasopressin 1b receptor (V1bR), that is preferentially expressed in α cells of both mouse and human pancreatic islets. Perifusion studies with mouse and human islets, including the use of V1bR-deficient mouse islets, clearly demonstrated that V1bR activation caused pronounced increases in glucagon release. Additional in vivo studies showed that V1bR-mediated stimulation of glucagon secretion plays an important role in the counterregulatory hyperglucagonemia triggered by insulin-induced hypoglycemia or glucopenic conditions (deficiency of intracellular glucose). Taken together, these potentially novel findings provide a rational basis for the development of new classes of clinically useful drugs able to selectively stimulate α cell G_q_ signaling under various pathophysiological conditions.

## Results

### Generation of mice expressing the GqD designer receptor selectively in pancreatic α cells.

In order to selectively stimulate G_q_ signaling in mouse α cells in vivo, we used a chemogenetic approach. Specifically, we expressed a G_q_-coupled DREADD (18) selectively in α cells of adult mice (α-GqD mice). We crossed mice harboring the *GqD* allele preceded by a *loxP-STOP-loxP* (*LSL*) sequence (*CAG-LSL-GqD* mice) (22) with *Gcg-CreERT2* mice (23). Following tamoxifen (TMX) treatment, Cre activity caused GqD expression in pancreatic α cells and endocrine L cells. Due to their relatively fast turnover, Cre-modified L cells have been shown to be replaced by wild-type (WT) L cells 4 weeks after TMX treatment (23). Four weeks after the last TMX injection, the mice expressed GqD selectively in α cells (hereafter referred to as α-GqD mice). TMX-injected *CAG-LSL-GqD* mice that did not carry the *Gcg-CreERT2* transgene and did not express the GqD receptor were used as control animals throughout the study.

**Figure 1:**
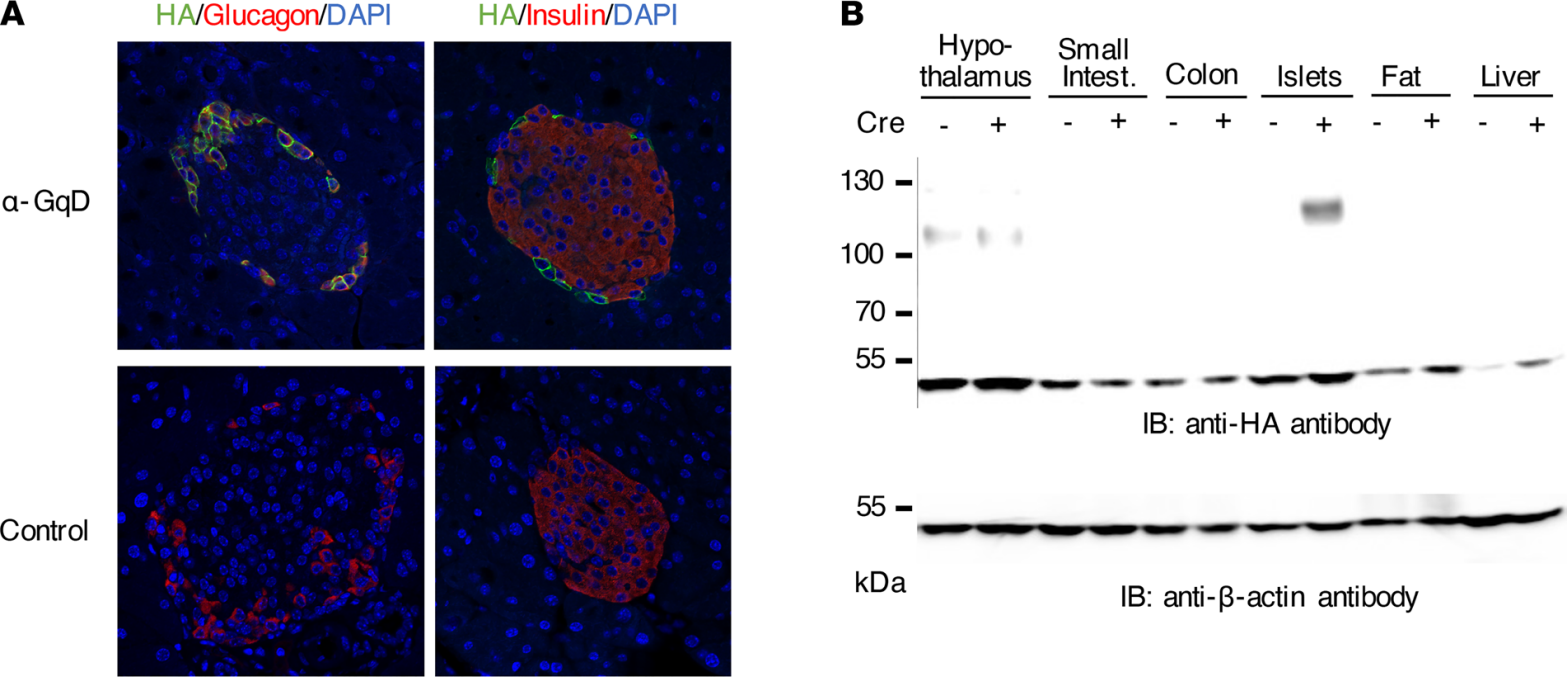
Selective expression of the GqD designer receptor in mouse pancreatic α cells. (**A**) Immunofluorescence staining of pancreatic slices from α-GqD mice and control littermates. The GqD receptor, which carried an HA epitope tag at its N-terminus (22), was detected with an anti-HA antibody (Alexa Fluor, green). Pancreatic α cells were stained with an anti-glucagon antibody (Alexa Fluor, red), and β cells were visualized with an anti-insulin antibody (Alexa Fluor, red). Nuclei were stained blue with DAPI mounting medium. (**B**) Western blot indicating that GqD expression is restricted to pancreatic islets of α-GqD mice. Cell lysates were prepared from the indicated tissues of α-GqD mice (+Cre) and control littermates (-Cre). Fat samples were prepared from epididymal white adipose tissue. Use of an anti-HA antibody showed that the GqD receptor was expressed only in pancreatic islets from α-GqD mice (band size: ~120 kDa). A nonspecific band (size ~50 kDa) band was observed in all lanes. Likewise, a nonspecific band was detectable with hypothalamic extracts prepared from both α-GqD and control mice (band size: ~110 kDa).

To confirm that the GqD receptor was selectively expressed in α cells of α-GqD mice, we carried out immunofluorescence studies using pancreatic sections from α-GqD and control mice. The GqD receptor was detected by using an antibody directed against the N-terminal HA tag fused to the N-terminus of GqD (22). Studies with pancreatic sections from α-GqD mice demonstrated that GqD was expressed by glucagon-expressing α cells but was undetectable in β cells and other cell types that did not express glucagon (Figure 1A). As shown in Figure 1A, essentially all glucagon-positive islet cells expressed the GqD receptor. We found that 97.5% of α cells expressed the GqD designer receptor. As expected, no GqD signal was detected in pancreatic sections prepared from control mice (Figure 1A). Immunoblotting studies confirmed that GqD expression was restricted to pancreatic islets when tissues were collected 4 weeks after the last TMX injection (Figure 1B). Importantly, no GqD expression was detected in the small intestine and colon, which contain proglucagon-producing L cells.

### Acute stimulation of α cell G_q_ signaling enhances glucagon secretion in vivo.

**Figure 2:**
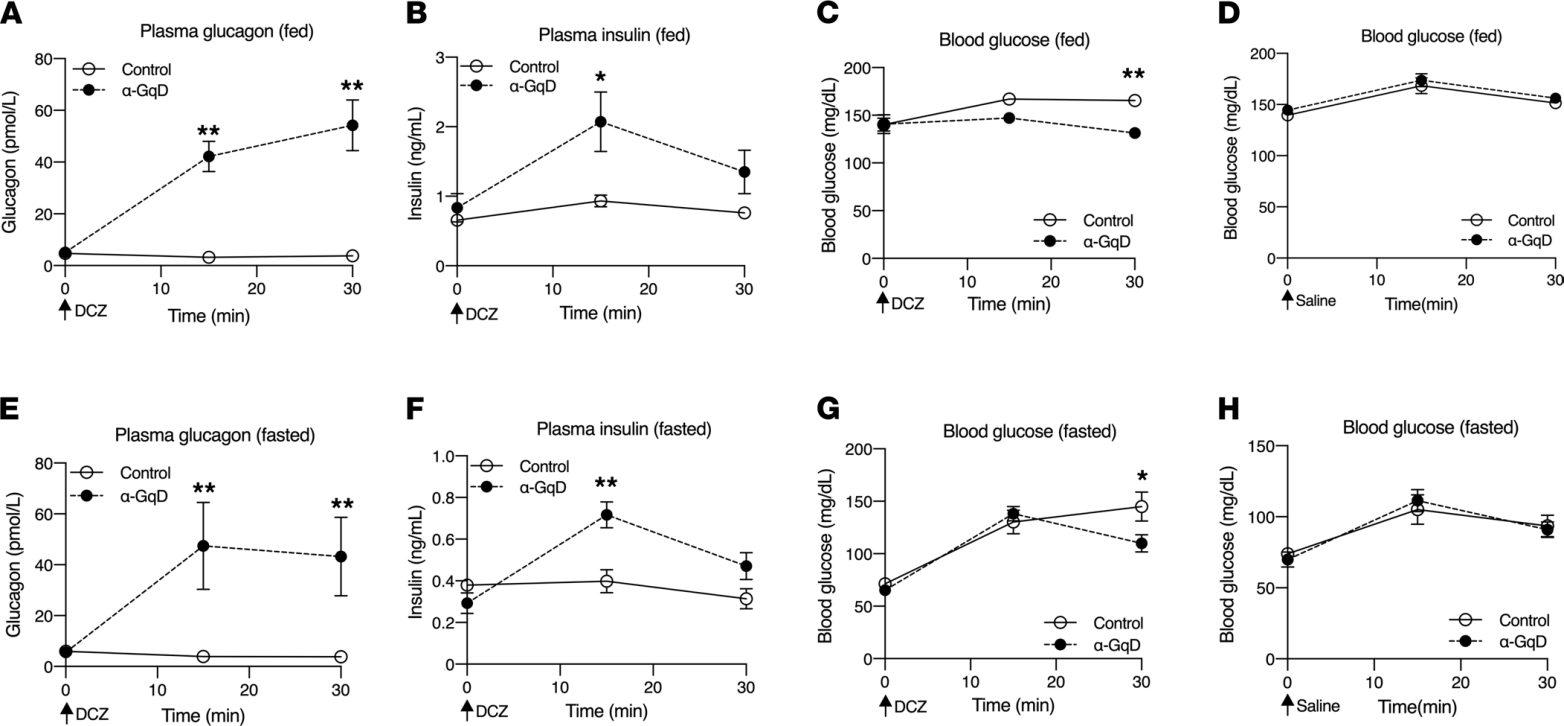
Acute activation of α cell G_q_ signaling stimulates glucagon and insulin secretion in vivo. α-GqD mice and control littermates (males) received a single i.p. dose of DCZ (10 μg/kg) (**A**–**C** and **E**–**G**) or vehicle (saline) (**D** and **H**), followed by the measurement of plasma glucagon (**A** and **E**), plasma insulin (**B** and **F**), and blood glucose levels (**C**, **D**, **G**, and **H**) under fed or fasting (~14-hour overnight fast) conditions. Blood samples were collected from the tail vein at the indicated time points. Data are given as mean ± SEM (α-GqD: *n* = 7; control: *n* = 8). *P ≤ 0.05 and **P ≤ 0.01 (mixed effects repeated measures ANOVA for after injection differences).

α-GqD mice and their control littermates (males) did not show any differences in body weight (Supplemental Figure 1A; supplemental material available online with this article; https://doi.org/10.1172/jci.insight.152852DS1). We first investigated whether DCZ (GqD agonist) (21) treatment of α-GqD mice was able to modulate glucagon release in vivo. In the absence of DCZ, α-GqD and control mice showed comparable plasma glucagon, plasma insulin, and blood glucose levels under both fed and fasting conditions (Figure 2, time 0). Treatment of α-GqD mice with a single dose of DCZ (10 μg/kg i.p.) caused a pronounced increase in plasma glucagon levels in both freely fed and fasted mice throughout the 30-minute observation period, as compared with control littermates (Figure 2, A and E). During this period, plasma insulin levels were also selectively elevated in DCZ-treated α-GqD mice (Figure 2, B and F). DCZ treatment of control mice resulted in a small increase in blood glucose levels, most likely due to the injection stress (Figure 2, C and G). This modest hyperglycemic effect was significantly reduced in DCZ-treated α-GqD mice (Figure 2, C and G), most likely due to the increase in plasma insulin levels caused by α cell G_q_ activation. This effect was not observed when α-GqD and control mice were injected with vehicle (saline) (Figure 2, D and H).

Acute DCZ (10 μg/kg i.p.) treatment of α-GqD or control mice had little or no effect on the plasma levels of the 2 major incretin hormones, GLP-1 and glucose-dependent insulinotropic polypeptide (GIP) (Supplemental Figure 1).

We also studied plasma hormone levels in female α-GqD and control mice. As observed with their corresponding male counterparts, acute DCZ (10 μg/kg i.p.) treatment of female α-GqD mice led to a striking elevation of plasma glucagon levels in both fed and fasted mice (Supplemental Figure 2). This response was associated with a significant increase in plasma insulin levels and improved glucose tolerance (Supplemental Figure 2). Following coinjection (i.p.) of DCZ and insulin, female α-GqD mice and control littermates showed similar insulin tolerance (Supplemental Figure 2). These observations indicate that the in vivo metabolic changes caused by activation of α cell G_q_ signaling are sex independent.

### Acute activation of α cell G_q_ signaling improves glucose tolerance.

**Figure 3:**
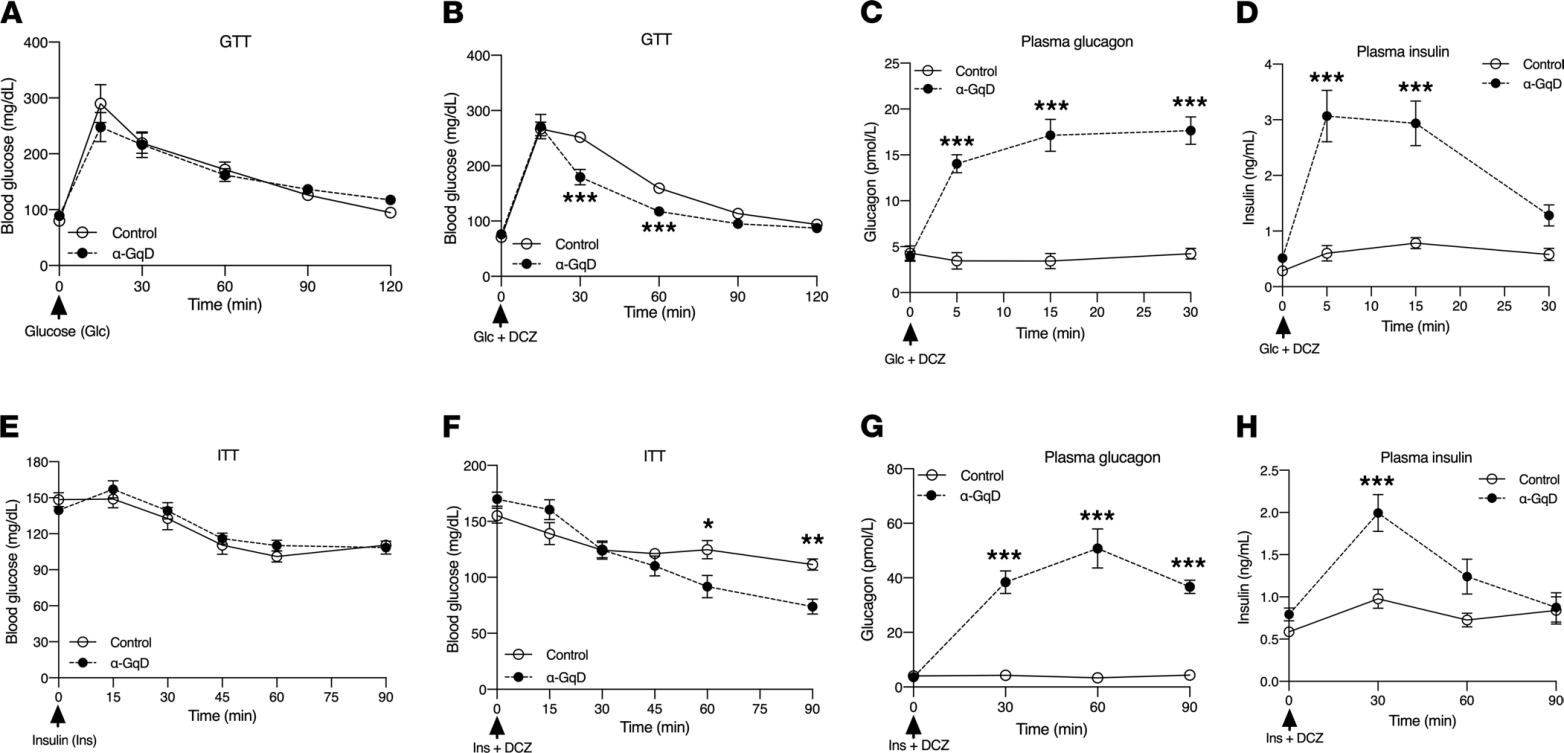
Acute stimulation of α cell G_q_ signaling improves glucose homeostasis. Metabolic studies were carried out with α-GqD mice and control littermates. (**A** and **B**) Glucose tolerance tests (GTT). Mice that had been fasted overnight were injected with glucose (2 g/kg i.p.), either in the absence (**A**) or presence (**B**) of DCZ (10 μg/kg i.p.). (**C** and **D**) Plasma glucagon (**C**) and plasma insulin (**D**) levels following coinjection of mice with glucose (2 g/kg i.p.) and DCZ (10 μg/kg i.p.) after fasting for 14 hours. (**E** and **F**) Insulin tolerance test (ITT). Mice that had been fasted 4 hours were injected with insulin (0.75 U/kg i.p.), either in the absence (**E**) or presence (**F**) of DCZ (10 μg/kg i.p.). (**G** and **H**) Plasma glucagon (**G**) and plasma insulin (**H**) levels following coinjection of mice with insulin (0.75 U/kg i.p.) and DCZ (10 μg/kg i.p.) after fasting for 4 hours. Blood samples were collected from the tail vein at the indicated time points. All experiments were carried out with male littermates (age: 16–20 weeks). Data are given as mean ± SEM (α-GqD: *n* = 8; control: *n* = 7). *P ≤ 0.05, **P ≤ 0.01, and ***P ≤ 0.001 (mixed effects repeated measures ANOVA for after injection differences).

We next subjected α-GqD mice and their control littermates (males) to an i.p. glucose tolerance test (GTT) (Figure 3). Injection of α-GqD mice and control littermates with glucose alone (2 g/kg i.p.) caused similar increases in blood glucose levels (Figure 3A). In contrast, i.p. coinjection of α-GqD mice with a mixture of glucose (2 g/kg) and DCZ (10 μg/kg) resulted in a significant improvement in glucose tolerance, as compared with control littermates coinjected in a similar fashion (Figure 3B). During the GTT, we also monitored changes in plasma glucagon and insulin levels. We found that coinjection of α-GqD mice with glucose and DCZ triggered very pronounced increases in both plasma glucagon and insulin levels (Figure 3, C and D). These data suggest that the improved glucose tolerance displayed by the coinjected α-GqD mice is most likely caused by increased insulin secretion following activation of α cell G_q_ signaling.

An ITT showed that treatment of α-GqD mice and control mice with insulin alone (0.75 U/kg i.p.) caused comparable decreases in blood glucose levels (Figure 3E). Strikingly, coinjection of insulin (0.75 U/kg i.p.) with DCZ (10 μg/kg i.p.) resulted in a significant increase in insulin sensitivity at later time points (60 and 90 minutes postinjection) (Figure 3F). As observed in the GTT experiments, coinjection of α-GqD mice with insulin and DCZ resulted in greatly increased plasma glucagon and insulin levels, as compared with control mice coinjected in the same fashion (Figure 3, G and H). Recent work suggests that glucagon release from pancreatic α cells is required for efficient release of insulin from β cells when glucose concentrations are high (7–9, 11). On the basis of these studies, it is likely that enhanced insulin secretion caused by activation of α cell G_q_ signaling is responsible for the improved glucose homeostasis displayed by the DCZ-treated α-GqD mice.

### Studies with perifused pancreatic islets prepared from α-GqD mice.

**Figure 4:**
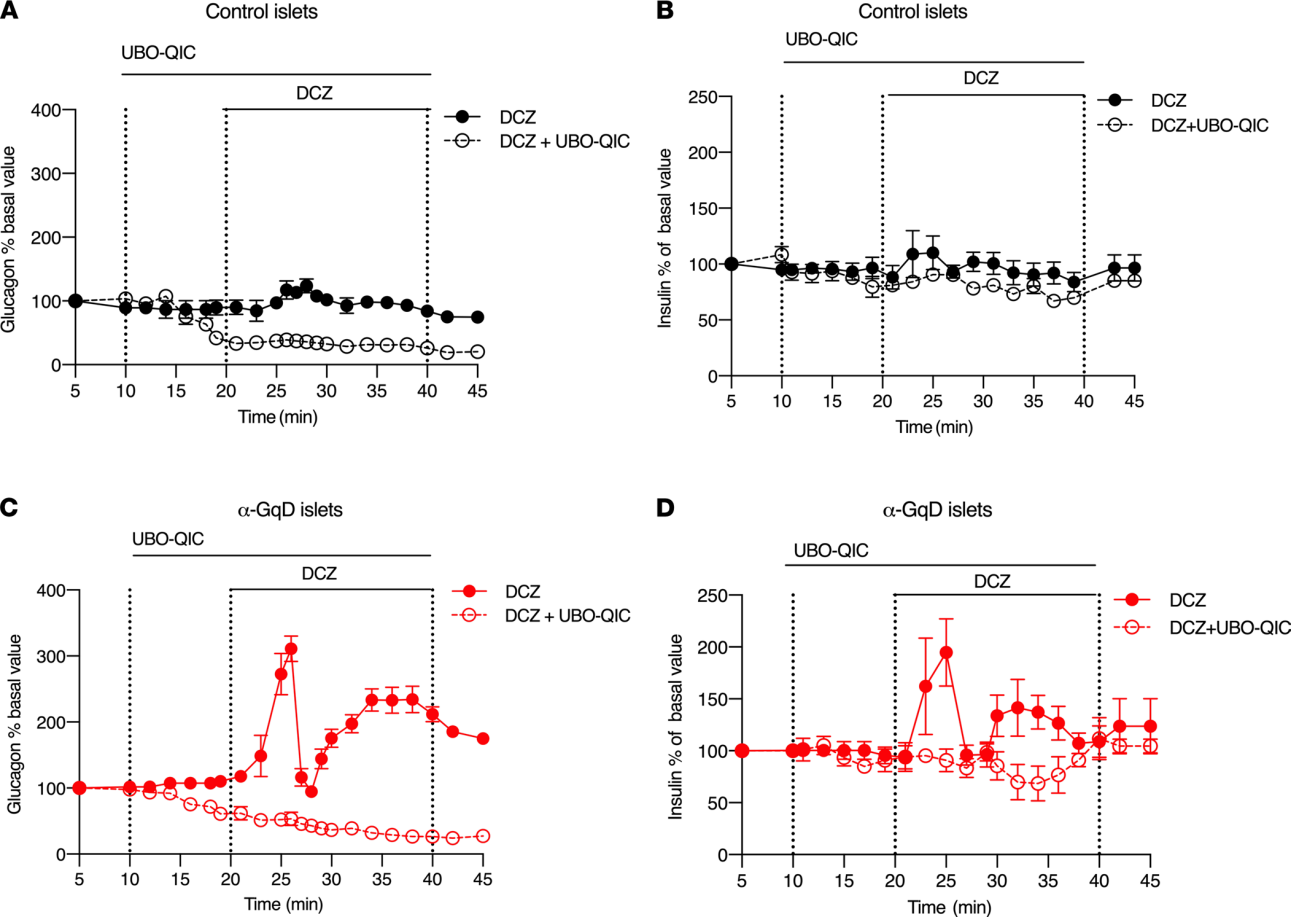
Activation of α cell G_q_ signaling stimulates glucagon and insulin secretion in isolated islets. (**A**–**D**) Perifused pancreatic islets prepared from control mice (**A** and **B**) or α-GqD littermates (**C** and **D**) were perifused with 3 mM glucose and DCZ (10 nM), either in the absence or presence of UBO-QIC (G_q_ inhibitor; 0.5 μM). Mouse islets were prepared from 16- to 20-week-old male and female mice. Data are given as mean ± SEM (4–6 perifusions with 75–100 islets per perifusion chamber).

To confirm that the in vivo metabolic changes observed with α-GqD mice were indeed due to altered G_q_ signaling in pancreatic α cells, we carried out a series of perifusion experiments using pancreatic islets prepared from α-GqD mice (α-GqD islets) and control littermates (control islets). The addition of DCZ (10 nM) had little or no effect on glucagon and insulin secretion from control islets (Figure 4, A and B) (glucose concentration: 3 mM). In contrast, under the same experimental conditions, DCZ treatment of α-GqD islets led to pronounced, biphasic increases of both glucagon and insulin secretion (Figure 4, C and D). Incubation of α-GqD islets with FR900359 (UBO-QIC; 0.5 μM), a selective G_q_ inhibitor (24), completely blocked the stimulatory effects of DCZ on glucagon and insulin secretion (Figure 4, C and D), confirming that the GqD-mediated effects on hormone secretion were indeed mediated by G_q_-type proteins. Taken together, the hormone release data obtained with α-GqD islets are in very good agreement with the in vivo metabolic phenotypes displayed by DCZ-treated α-GqD mice (Figures 2 and 3).

It should also be noted that UBO-QIC treatment lowered glucagon secretion below baseline levels in both control and α-GqD islets (Figure 4, A and C). This observation suggests that G_q_-type G proteins are tonically active in mouse α cells, thus contributing to proper basal glucagon release.

### Chronic activation of α cell G_q_ signaling improves glucose homeostasis in obese mice.

To test the potential translational relevance of the metabolic improvements observed after activation of α cell G_q_ signaling, we maintained α-GqD and control littermates on a high-fat diet (HFD) for at least 6 weeks. This calorie-rich diet is known to cause obesity, glucose intolerance, and several other metabolic changes that are key features of human T2D (25). To examine the effects of chronic activation of α cell G_q_ signaling on glucose homeostasis, all mice received daily injections of DCZ (10 μg/kg i.p.) for at least 7 days (26). Studies with other α cell–specific DREADD mouse strains showed that DCZ (10 μg/kg i.p.) affects glucagon release for at least 4–6 hours (LL, unpublished observations). HFD α-GqD mice and control littermates did not show any differences in body weight or food intake during the DCZ injection period (Supplemental Figure 3, A and B). However, daily DCZ treatment of α-GqD mice, but not of control littermates, resulted in significantly increased plasma glucagon and insulin levels under both fed and fasted conditions throughout the entire DCZ administration period (Supplemental Figure 4, A, B, D, and E). On day 1, DCZ injection led to elevated blood glucose levels in α-GqD mice, but not in control littermates, measured 1 hour after drug injection (Supplemental Figure 4C). After 7 days of DCZ treatment, glucose levels were similar between the 2 groups of mice (Supplemental Figure 4, C and F). For this reason, we performed all metabolic studies after 7 days of DCZ treatment.

**Figure 5:**
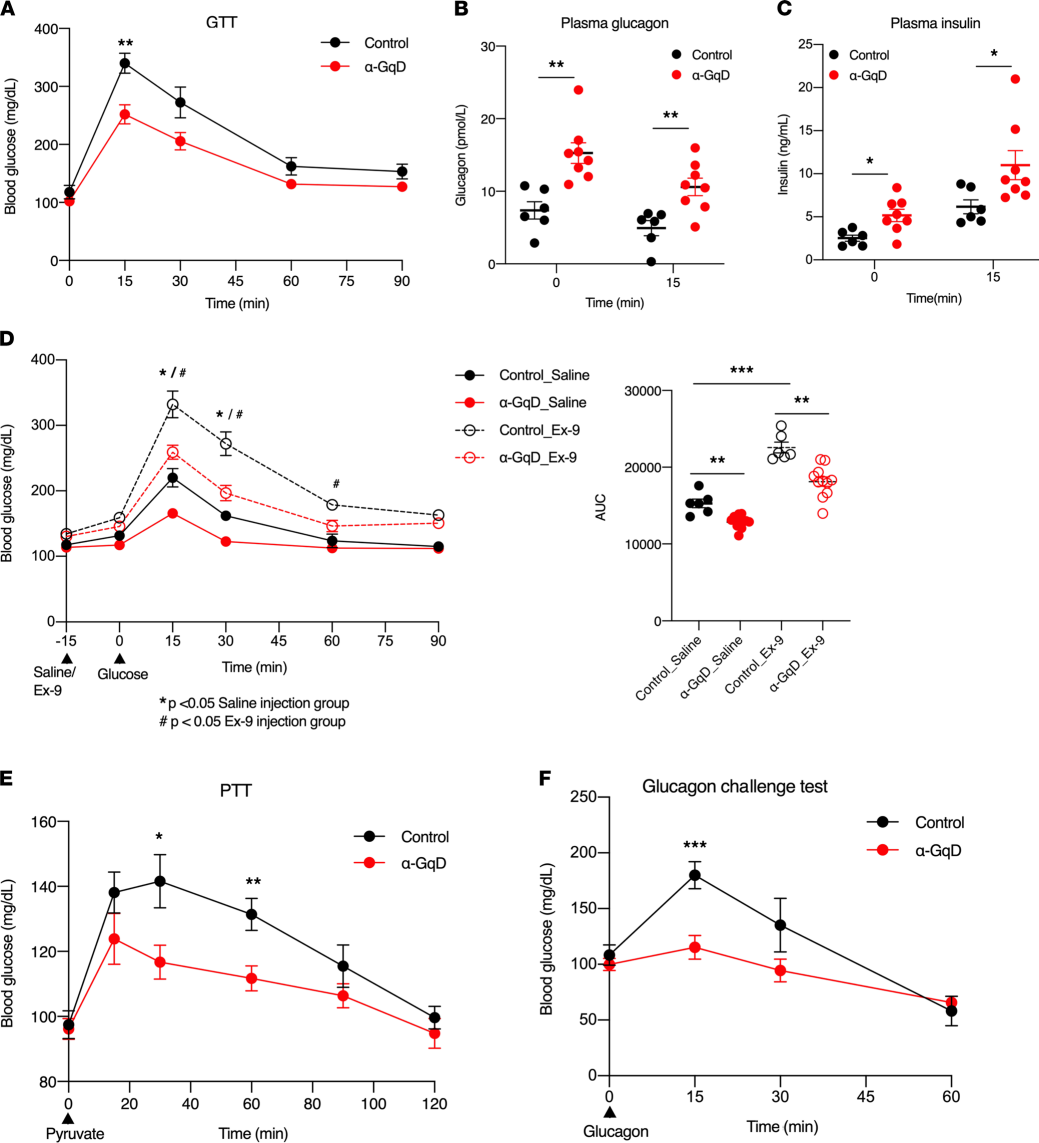
Chronic activation of α cell Gq signaling improves glucose homeostasis in obese α-GqD mice. α-GqD mice and control littermates were maintained on a high-fat diet for at least 6 weeks. Metabolic studies were carried out with mice that had received daily injections of DCZ (10 μg/kg i.p.) for 27 days. (**A**) Glucose tolerance test carried out with overnight-fasted mice. Glucose was administered (1 g glucose/kg i.p.) 1 hour after the last DCZ injection. (**B** and **C**) Plasma glucagon (**B**) and insulin (**C**) levels of fasted mice prior to (time 0) and 15 minutes after glucose injection (1 g/kg i.p.). (**D**) Glucose tolerance test (see **A** for details) carried out with mice injected with exendin(9-39) (Ex-9, 50 mg/mouse) or vehicle (saline) prior to glucose treatment. The panel to the right shows area under the curve (AUC) for each mouse (arbitrary units). (**E**) Pyruvate tolerance test (PTT) (1 g/kg i.p.) performed with overnight-fasted mice. (**F**) Glucagon challenge test (100 μg/kg i.p.) carried out with mice fasted for 6 hours. All experiments were carried out with male littermates that were about 35 weeks old. Data are given as mean ± SEM (α-GqD: *n* = 8–11; control: *n* = 5–10). *P ≤ 0.05, **P ≤ 0.01, and ***P ≤ 0.001 (mixed effects repeated measures ANOVA for after injection differences).

After 1 week of DCZ treatment, all mice were subjected to an i.p. GTT. We found that α-GqD mice displayed a significant improvement in glucose tolerance, as compared with control littermates (Figure 5A). Prior to glucose administration (1 g/kg i.p.), plasma glucagon and insulin levels were significantly higher in the DCZ-treated α-GqD mice than in the DCZ-treated control animals (Figure 5, B and C). Plasma glucagon and insulin levels remained significantly elevated following glucose injection (Figure 5, B and C). These data indicate that chronic activation of α cell G_q_ signaling improves glucose homeostasis in obese, glucose-intolerant mice.

An insulin tolerance test showed that HFD α-GqD mice and control littermates chronically treated with DCZ did not differ in insulin sensitivity (Supplemental Figure 3C). To test the hypothesis that elevated insulin levels caused by glucagon activation of β cell GLP-1 receptors (7–9, 11) are responsible for the improved glucose tolerance observed with HFD α-GqD mice chronically treated with DCZ, we injected HFD α-GqD mice and control littermates with the selective GLP-1 receptor antagonist exendin(9-39) (Ex-9; 50 μg/mouse) (27, 28), prior to glucose administration. Following Ex-9 treatment, HFD α-GqD mice continued to show improved glucose tolerance (Figure 5D), suggesting that the beneficial metabolic effects caused by chronic activation of α cell G_q_ signaling were not due to glucagon-induced activation of β cell GLP-1 receptors. Interestingly, in a pyruvate challenge test, DCZ-treated HFD α-GqD mice showed significantly reduced blood glucose excursions, as compared with their HFD control littermates (Figure 5E), indicative of reduced hepatic in vivo gluconeogenesis. In agreement with these results, the ability of a glucagon bolus (100 μg/kg i.p.) to trigger hyperglycemia (glucagon challenge test) was completely abolished in DCZ-treated HFD α-GqD mice (Figure 5F). These observations strongly suggest that chronic elevation of plasma glucagon levels due to enhanced α cell G_q_ signaling resulted in hepatic glucagon resistance, providing an explanation for the improved glucose tolerance displayed by the DCZ-treated HFD α-GqD mice.

Liver weight, hepatic triglyceride content, and hepatic glucagon receptor expression levels did not differ significantly between the 2 groups of mice (Supplemental Figure 3, D–F). Taken together, these findings suggest that the glucagon resistance displayed by DCZ-treated HFD α-GqD mice may result from hepatic glucagon receptor desensitization.

### G_q_-coupled receptors endogenously expressed by α cells stimulate glucagon secretion in mouse and human islets.

The metabolic phenotypes DCZ-treated α-GqD mice displayed suggested that G_q_-coupled receptors endogenously expressed by α cells might regulate glucagon release in a similar fashion. To identify such receptors, we took advantage of published single-cell RNA-Seq (scRNA-Seq) data from human and mouse islets (human islets: Gene Expression Omnibus [GEO] GSE81608; mouse islets: GEO GSE80673) (29, 30). Based on this information, we compiled a list of G_q_-coupled receptors that are expressed at relatively high levels in mouse and/or human α cells (Supplemental Figure 5). Interestingly, the V1bR (*V1bR* or *Avpr1b*) is preferentially expressed by α cells in both mouse and human islets. To detect *V1bR* expression in mouse islets, we crossed *Avpr1b-Cre* knockin mice (31) with a mouse line that expresses the tdTomato reporter protein in a Cre-dependent fashion (see Supplemental Figure 6 for details). In agreement with the gene expression data, immunofluorescence staining of mouse islets showed that the V1bR subtype was selectively expressed by mouse α cells (Supplemental Figure 6). Virtually all glucagon-positive cells expressed *V1bR* (Supplemental Figure 6).

**Figure 6:**
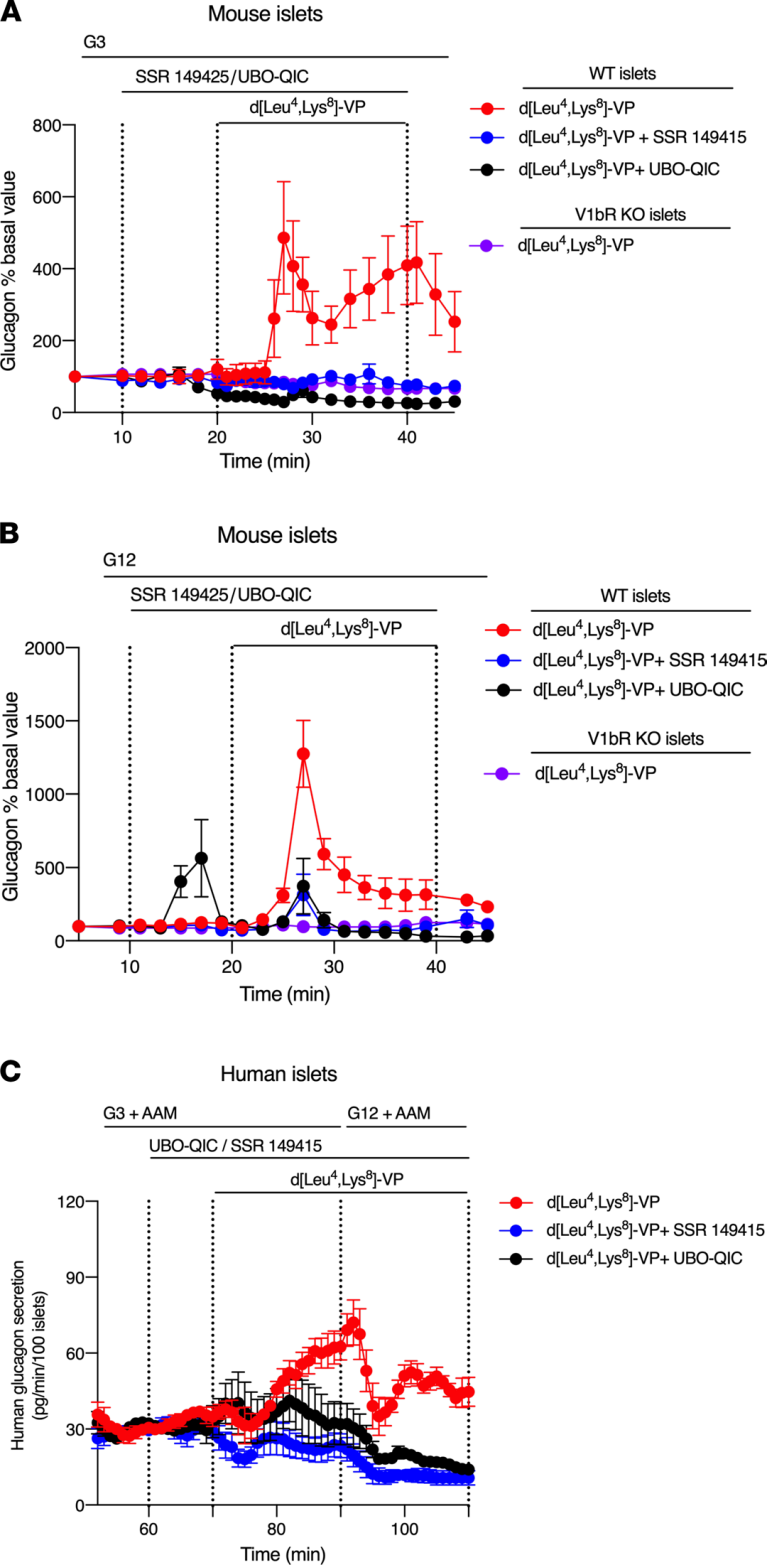
Activation of the V1bR subtype stimulates glucagon secretion in mouse and human islets. (**A** and **B**) Studies with perifused mouse islets. Mouse islets were prepared from male mice (age: 12–20 weeks). Islets from WT mice were perifused with 3 mM glucose (G3) (**A**) or 12 mM glucose (G12) (**B**) in the presence of 1 nM d[Leu^4^, Lys^8^]-VP (V1bR-selective agonist) (**A** and **B**). The stimulatory effect of d[Leu^4^, Lys^8^]-VP on glucagon secretion was blocked by 1 μM SSR149415 (V1bR antagonist) (**A** and **B**). The d[Leu^4^, Lys^8^]-VP–induced glucagon responses were abolished in the presence of 0.5 μM UBO-QIC, a selective G_q_ inhibitor (**A** and **B**). Moreover, d[Leu^4^, Lys^8^]-VP (1 nM) treatment of islets derived from mice lacking functional V1bRs (*Avpr1b-Cre+/+* knockin mice; alternative name: V1bR-KO mice) had no effect on glucagon release (**A** and **B**). (**C**) Studies with human islets. Islets from human donors were perifused with physiological amino acid mixture (AAM) and G3 or 16.7 mM of glucose (G16.7), respectively, in the presence of d[Leu^4^, Lys^8^]-VP (V1bR agonist; 1 nM). The stimulatory effect of d[Leu^4^, Lys^8^]-VP on glucagon secretion was blocked by SSR149415 (50 nM) or by UBO-QIC (1 μM). Data are given as mean ± SEM (3–6 perifusions with 75–100 islets per perifusion chamber per group).

To investigate the ability of α cell V1bRs to promote glucagon secretion, we treated perifused WT islets with a selective V1bR agonist (d[Leu^4^,Lys^8^]-VP; 1 nM) (32). As expected, addition of the V1bR agonist led to a robust, biphasic increase in glucagon secretion (glucose concentration: 3 mM) (Figure 6A), a pattern similar to that observed with DCZ-treated α-GqD islets. This stimulatory effect on glucagon secretion was also observed when glucose levels were raised to 12 mM (Figure 6B). The V1bR agonist–induced increase in glucagon release was prevented by treatment of WT islets with a selective V1bR antagonist (SSR149415; 1 μM) (33), confirming the involvement of V1bR in this response (Figure 6, A and B). Likewise, inhibition of G_q_ signaling by UBO-QIC (0.5 μM) also abolished the ability of the V1bR agonist to promote glucagon release from WT islets (Figure 6, A and B). Finally, d[Leu^4^, Lys^8^]-VP (1 nM) treatment of islets derived from mice lacking functional V1bRs (*Avpr1b-Cre+/+* knockin mice; alternative name: V1bR-KO mice) (31) had no effect on glucagon release (Figure 6, A and B). Taken together, these data clearly indicate that activation of islet (α cell) V1bRs promotes glucagon release via activation of G_q_-type G proteins.

We next carried out glucagon secretion studies using islets from human donors. In agreement with the data obtained with mouse islets, d[Leu^4^,Lys^8^]-VP (1 nM; V1bR agonist) stimulated the secretion of glucagon from human islets in a G_q_-dependent manner (Figure 6C). The glucagon response to d[Leu^4^,Lys^8^]-VP was absent in the presence of SSR149415 (50 nM; V1bR antagonist) (Figure 6C). These data strongly support the concept that stimulation of the V1bR subtype expressed by human α cells stimulates the release of glucagon.

d[Leu^4^,Lys^8^]-VP was able to promote insulin release in some but not all human islet preparations. As a result, d[Leu^4^,Lys^8^]-VP–mediated increases in glucose-stimulated insulin secretion failed to reach statistical significance (Supplemental Figure 7).

Possible explanations for this data scatter are that the different islet batches were collected under somewhat different conditions or that the health status of the islet donors affected the outcome of the insulin secretion studies.

### V1bRs contribute to hypoglycemia/glucopenia-induced glucagon release in vivo.

**Figure 7:**
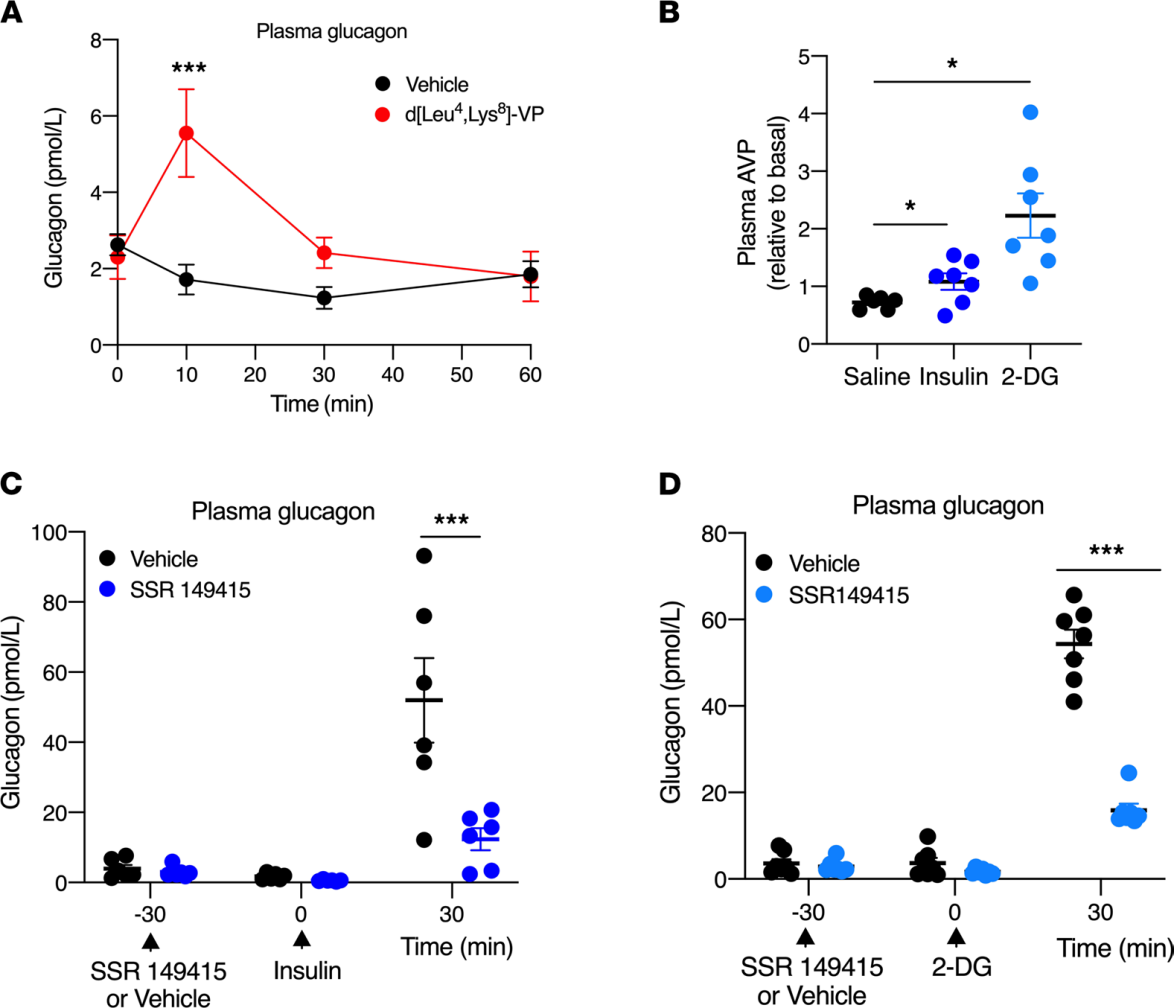
V1bRs contribute to hypoglycemia/glucopenia-induced glucagon release in vivo. (**A**) V1bR-mediated increase in glucagon release in vivo. WT mice received a single dose of d[Leu^4^, Lys^8^]-VP (V1bR agonist; 0.3 μg/kg i.v.), followed by the measurement of plasma glucagon levels. (**B**) Increases in plasma AVP levels evoked by insulin and 2-deoxy-d-glucose (2-DG). WT mice were injected i.p. with saline, insulin (0.75 U/kg), or 2-DG (500 mg/kg). Plasma AVP levels were measured immediately before and 30 minutes after injections. Data are presented as plasma AVP levels relative to preinjection values. (**C** and **D**) Hypoglycemia/glucopenia-induced glucagon release is inhibited by a V1bR antagonist. WT mice were injected i.p. with either vehicle or SSR149415 (25 mg/kg), a selective V1bR antagonist. Thirty minutes later, the mice were treated i.p. with either insulin (1.25 U/kg) (**C**) or 2-DG (500 mg/kg) (**D**). Plasma glucagon levels were determined immediately prior to or 30 minutes after insulin or 2-DG treatment. Male mice (age: 8–12 weeks) were used for this study. Data are given as mean ± SEM (*n* = 6 or 7 mice per group). *P ≤ 0.05 and ***P ≤ 0.001 (mixed effects repeated measures ANOVA for after injection differences).

We next examined whether V1bR-mediated glucagon release from mouse α cells is of physiological relevance under in vivo conditions. Specifically, we injected WT mice with d[Leu^4^,Lys^8^]-VP (0.3 μg/kg; V1bR agonist) via the tail vein (32). The V1bR agonist stimulated glucagon secretion at an early time point (10 minutes) but not at later postinjection times (Figure 7A), probably due to the limited metabolic stability of this peptide agonist (note that the parent compound arginine vasopressin, AVP, has a plasma half-life of only 15–20 minutes). However, the V1bR agonist had little or no effect on blood glucose and plasma insulin levels (Supplemental Figure 8, A and B), perhaps due to enhanced V1bR activity in other peripheral tissues (34).

It is well-known that the plasma levels of AVP, the endogenous V1bR agonist, are regulated by circulating blood glucose levels. Preclinical and clinical studies have shown that insulin-induced hypoglycemia leads to increased plasma AVP levels (35–38). As expected, treatment of WT mice with insulin (0.75 U/kg i.p.) caused a significant increase in plasma AVP levels (Figure 7B).

To explore whether endogenous AVP/V1bR signaling contributed to hypoglycemia-induced glucagon secretion, we injected WT mice with a selective V1bR antagonist (SSR149415; 25 mg/kg, i.p.) (33) 30 minutes prior to insulin administration. SSR149415 treatment abolished insulin-induced glucagon secretion (Figure 7C), showing that intact AVP/V1bR signaling is required for efficient glucagon release following insulin-induced hypoglycemia. SSR149415 treatment had no significant effect on the magnitude of the hypoglycemic effect caused by insulin administration (Supplemental Figure 8C). One possible explanation for this observation is that the insulin injection resulted in supraphysiological insulin levels that masked the hyperglycemic effect predicted to occur after V1bR-induced glucagon secretion.

The glucose analog, 2-DG, a competitive inhibitor of the production of glucose-6-phosphate from glucose, interferes with glucose metabolism, resulting in intracellular glucopenia in the brain and other tissues (39–42). Previous studies have shown that 2-DG treatment of human volunteers or rodents leads to elevated AVP levels (43, 44) and a counterregulatory stimulation of glucagon secretion (40, 42). We therefore examined whether endogenous AVP/V1bR signaling contributes to 2-DG–evoked increases in glucagon secretion. Strikingly, 2-DG (500 mg/kg, i.p.) treatment of WT mice resulted in a pronounced increase in both plasma AVP and plasma glucagon levels (Figure 7, B and D). The 2-DG–evoked elevation in plasma glucagon levels was greatly reduced after pretreatment of WT mice with SSR149415 (V1bR antagonist, 25 mg/kg, i.p.; Figure 7D).

Taken together, these data clearly indicate that endogenous AVP/V1bR signaling makes a critical contribution to the increase in glucagon secretion following hypoglycemic and glucopenic conditions.

## Discussion

Recent advances in the glucagon/α cell field have led to renewed interest in the role of glucagon as a key regulator of glucose homeostasis. Several recent in vitro and in vivo studies demonstrated that glucagon release from α cells can act in a paracrine fashion to promote insulin release from β cells and that this activity is required for maintaining euglycemia in animal models (7–11). In line with these observations, dual agonists that stimulate both GLP-1 and glucagon receptors are currently under development for the therapy of T2D and related metabolic disorders (12, 13).

Since GPCRs represent excellent drug targets (15), a better understanding of how α cell function and glucagon release are regulated by distinct GPCR/G protein signaling pathways is of considerable potential translational relevance. Like other cell types, including pancreatic β cells (14, 45), α cells express many GPCRs that are linked to the activation of G_q_-type G proteins (G_q_/G_11_) (14). To explore the in vivo metabolic consequences of selectively activating G_q_ signaling in α cells, we generated mice that expressed a G_q_-coupled designer GPCR selectively in α cells (α-GqD mice) (Figure 1).

Strikingly, acute treatment of α-GqD mice with DCZ, a CNO derivative with increased potency at DREADD designer receptors (21), resulted in elevated plasma glucagon levels that were accompanied by increased plasma insulin levels (Figure 2). DCZ treatment of α-GqD mice also led to improved glucose tolerance (Figure 3). Moreover, chronic CNO treatment of obese, glucose-intolerant α-GqD mice led to greatly improved glucose homeostasis (Figure 5), indicating that α cell G_q_ signaling represents a potential therapeutic target since this pathway is not subject to desensitization.

In agreement with the in vivo metabolic phenotypes displayed by the α-GqD mice, DCZ treatment of isolated islets prepared from α-GqD mice stimulated both glucagon and insulin release (Figure 4). DCZ treatment of α-GqD islets increased glucagon and insulin release in a biphasic fashion (Figure 4). Since glucagon can directly stimulate somatostatin secretion under both low- and high-glucose conditions through glucagon and GLP-1 receptors expressed on δ cells (46), one possible explanation for this observation is that the initial peak in hormone release is counteracted by glucagon-induced somatostatin release from adjacent δ cells (14). However, this hypothesis needs to be tested experimentally.

Taken together, our in vivo and in vitro data support a model in which acute activation of α cell G_q_ signaling promotes the release of glucagon, which, via paracrine action on adjacent β cells (7–11), triggers the release of insulin.

It is well-known that agonist binding to G_q_-coupled receptors leads to the activation of different isoforms of phospholipase C, resulting in the breakdown of phosphatidyl inositol and the generation of the second messengers, inositol 1,4,5-trisphosphate (IP3) and diacylglycerol (16, 17). IP3 binding to specific receptors in the endoplasmic reticulum results in the release of Ca^2+^ from intracellular stores (16, 17). Several lines of evidence indicate that increases in intracellular Ca^2+^ levels in α cells are a major trigger for glucagon release (47). Based on these previous findings, it is highly likely that the G_q_-mediated increases in glucagon secretion observed in the present study depend on the ability of activated G_q_ to raise cytoplasmic Ca^2+^ levels in α cells.

Walker et al. (48) recently used an adenovirus-based approach to express the GqD designer receptor in genetically engineered human pseudoislets that resembled native human islets. CNO treatment of the GqD-expressing pseudoislets led to a robust increase in glucagon secretion. Although GqD expression was not restricted to α cells, this observation is consistent with the concept that stimulation of human α cell G_q_ signaling promotes glucagon secretion. Since the GqD construct was also expressed by β cells, it remains unclear to what extent CNO-induced glucagon release contributed to the CNO-evoked biphasic effects on insulin secretion in this experimental system (48).

By analyzing published scRNA-Seq data (29, 30), we identified a G_q_-coupled receptor, the V1bR, that is selectively expressed in both mouse and human α cells, as compared with β cells and other islet cells. Studies with isolated mouse and human islets showed that agonist stimulation of WT islets with a selective V1bR agonist caused a pronounced increase in glucagon release (Figure 6). V1bR agonist–induced glucagon responses could be prevented by pretreatment of islets with UBO-QIC (selective G_q_ inhibitor) (Figure 6), indicating that the V1bR-evoked effects on glucagon secretion were mediated by G_q_-type G proteins.

The V1bR is part of a family of receptors that is activated by AVP, a nonapeptide synthesized by distinct neuronal subpopulations of the hypothalamus (49). Other members of this family are the V1a and V2 receptor subtypes (34). Under physiological conditions, an increase in plasma osmolality represents the major trigger of AVP release, leading to antidiuresis via activation of V2 receptors expressed by kidney collecting duct cells (50). However, AVP secretion can also be induced by other factors in both animal models and humans, including hypoglycemic or glucopenic conditions (35–38, 43, 44). Interestingly, acute hypoglycemia has been shown to activate AVP-containing hypothalamic neurons in animal models (51, 52).

The V1bR is expressed in various regions of the brain and in several peripheral tissues (34). The best known function of the V1bR is to stimulate the release of ACTH from the pituitary gland (53). In contrast, little is known about the potential metabolic functions of this receptor subtype. In agreement with the hormone release data obtained with V1bR agonist-treated (d[Leu^4^,Lys^8^]-VP) WT islets (Figure 6), acute V1bR agonist treatment of WT mice resulted in a significant increase in plasma glucagon levels in vivo (Figure 7A). These data are in agreement with the concept that AVP-mediated increases in plasma glucagon levels involve the activation of α cell V1bRs.

Treatment with insulin and several other insulin secretagogues can cause hypoglycemia, which remains a major limiting factor in achieving euglycemia in patients with type 1 diabetes (T1D) or T2D (3, 4, 54). It has been estimated that about 5% of T1D patients and a significant number of T2D patients die from insulin-induced hypoglycemia (55). In both T1D and T2D, the ability of glucagon to counteract insulin-induced hypoglycemia has been shown to be impaired (3, 4, 14). For this reason, a better understanding of the cellular and molecular mechanisms that control glucagon release under hypoglycemic conditions is of considerable clinical relevance.

Based on the ability of V1bRs to promote glucagon release, we explored the possibility that V1bR signaling contributes to the counterregulatory increase in plasma glucagon levels triggered by insulin-induced hypoglycemia. In agreement with this concept, we demonstrated that treatment of WT mice with insulin caused a significant increase in plasma AVP levels (Figure 7B), consistent with previous findings (35–37). Strikingly, the insulin-evoked increase in plasma glucagon levels was abolished after pretreatment of WT mice with a selective V1bR antagonist (SSR149415; Figure 7C), indicating that AVP-dependent activation of V1bRs plays a key role in the compensatory increase in glucagon release following insulin-induced hypoglycemia.

During hypoglycemia, glucagon secretion also serves as feedback to protect the organism from the detrimental effects of low glucose levels in neurons and other cell types (glucopenia) (40, 42). In metabolism research, 2-DG, a competitive inhibitor of the production of glucose-6-phosphate from glucose, is often employed to induce glucopenia in the brain and other tissues (39–42). Similar to insulin-induced hypoglycemia, 2-DG administration leads to elevated AVP levels in both humans and experimental animals (43, 44). On the basis of these findings, we examined whether endogenous AVP/V1bR signaling contributes to 2-DG–induced glucagon release. In agreement with this concept, 2-DG treatment of WT mice resulted in a pronounced increase in both plasma AVP and plasma glucagon levels, and the 2-DG–evoked increase in glucagon secretion was greatly reduced after pretreatment with a V1bR antagonist (Figure 7, B and D).

Glucagon release from pancreatic α cells is known to be regulated by several other GPCRs endowed with distinct G protein–coupling properties (14). For example, it is well-known that δ cell–derived somatostatin inhibits glucagon release in a paracrine fashion via activation of G_i_-coupled somatostatin receptors expressed by α cells (56). On the other hand, following a meal, the incretin GIP stimulates glucagon release by binding to α cell GIP receptors, which preferentially interact with G_s_ (57).

In conclusion, by using a combination of different experimental approaches, we demonstrated that activation of α cell G_q_ signaling results in enhanced glucagon secretion and that this effect triggers multiple beneficial metabolic effects in both lean and obese, glucose-intolerant mice. We also identified an endogenous G_q_-coupled receptor, the V1bR subtype, that is enriched in mouse and human α cells. As expected, agonist activation of the V1bR promoted glucagon release in a G_q_-dependent fashion. Finally, we demonstrated that endogenous AVP/V1bR signaling plays a key role in mediating counterregulatory glucagon secretion under hypoglycemic and glucopenic conditions. Given the central role of glucagon in regulating glucose homeostasis, these potentially novel findings should be of considerable translational relevance.

## Methods

### Drugs, reagents, and mouse models.

All drugs, reagents, antibodies, and mouse models used are listed in Supplemental Table 1.

### Mouse maintenance.

Mice were kept at 23°C in a specific pathogen–free facility with a 12-hour light/12-hour dark cycle, with unrestricted access to water and standard rodent chow (7022 NIH-07 diet, 15% kcal fat, energy density 3.1 kcal/g, Envigo Inc.). In a subset of experiments, mice (age: 16 weeks) were maintained on an HFD (F3282, 60% kcal fat, energy density 5.5 kcal/g, Bioserv) for at least 6 weeks. For chronic DCZ treatment experiments, HFD mice received daily injections of DCZ (10 μg/kg) for at least 7 days. Unless stated otherwise, adult male littermates were used for all experiments. The WT mice used for some of the studies were obtained from Taconic (C57BL/6NTac mice).

### Generation of α-GqD mice and other mutant mouse strains.

Mutant mice expressing the GqD designer receptor (18) selectively in α cells (α-GqD mice) were generated by crossing *CAG-LSL-GqD* mice (alternative name: *CAG-LSL-hM3Dq* mice) (22) with *Gcg-CreERT2* knockin mice, which harbor a tamoxifen-inducible form of Cre recombinase (23). In order to induce nuclear Cre activity, we injected mice carrying 1 copy of *Gcg-CreERT2* and 1 copy of *CAG-LSL-GqD* for 6 consecutive days with TMX suspended in corn oil (2 mg TMX i.p. per mouse per day). As outlined in detail under Results, this experimental protocol led to the selective expression of GqD in pancreatic α cells. For control purposes, we injected *CAG-LSL-GqD* mice that did not carry the *Gcg-CreERT2* transgene with TMX in an identical fashion. These mice did not express the GqD receptor and served as control animals throughout the study. All animals used were maintained on a C57BL/6 background.

*Avpr1b-Cre+/+* knockin mice (genetic background: C57BL/6) were a gift from W. Scott Young (National Institute of Mental Health, NIH, Bethesda, Maryland, USA) (31). These mice lack functional V1bRs due to the insertion of the *Cre* transgene.

### Mouse genotyping.

Mouse tail DNA was used for PCR genotyping of *Gcg-CreERT2* and *CAG-LSL-GqD* mice. To detect the *Gcg-CreERT2* transgene, the following PCR primer pair was used: Cre-F, 5′-CCTGGAAAATGCTTCTGTCCG-3′; Cre-R, 5′-CAGGGTGTTATAAGCAATCCC-3′) (product size: 400 bp). The presence of the *CAG-LSL-GqD* allele was confirmed by the use of the following primers: GqD-F, 5′-CGCCACCATGTACCCATAC-3′; GqD-R, 5′-GTGGTACCGTCTGGAGAGGA-3′ (product size: 204 bp). PCR reactions were carried out using standard procedures.

### Determination of blood glucose levels and plasma hormone measurements.

Blood for glucose and plasma hormone measurements was collected from the mouse tail vein. Blood glucose levels were measured with an automated blood glucose reader (Glucometer Elite Sensor; Bayer). Blood samples used for plasma hormone measurements were collected in EDTA-coated tubes (SAFE-T-FILL, RAM Scientific) containing aprotinin (500 KIU/mL) and dipeptidyl peptidase-4 inhibitor (KR-62436, 0.01 mM). Plasma was obtained by centrifugation at 10,000*g* for 10 minutes at 4°C. Plasma insulin, glucagon, GLP-1, GIP, and AVP levels were determined using commercially available ELISA kits (see Supplemental Table 1 for details).

### DCZ challenge test.

To assess the in vivo effects of acute activation of the GqD receptor in α cells, α-GqD mice and their control littermates were injected with DCZ (10 μg/kg i.p. in saline). DCZ was administered to mice that had free access to food or that had been fasted overnight for about 14 hours. Blood glucose concentrations and plasma insulin and glucagon levels were determined using blood collected from the tail vein at defined time points.

### Treatment of WT mice with a selective V1bR agonist.

WT mice (C57BL/6NTac mice, 12-week-old males) were injected i.v. (via the tail vein) with either vehicle (saline) or d[Leu^4^,Lys^8^]VP (0.3 μg/kg), a selective V1bR agonist (32). Blood was collected from the tail vein for blood glucose and plasma glucagon and insulin measurements (see above) immediately prior to injections and 10, 30, and 60 minutes afterwards.

### GTT and glucose-stimulated insulin secretion.

To evaluate the effects of activation of G_q_ signaling in α cells on glucose tolerance, α-GqD mice and control littermates were fasted overnight and then injected with glucose (2 g/kg i.p. for chow diet mice; 1 g/kg i.p. for HFD mice), either in the presence or the absence of DCZ (10 μg/kg i.p. in saline). Blood glucose concentrations were determined immediately prior to injections and 15, 30, 60, 90, and 120 minutes afterward. In a subset of studies, mice were injected i.p. with Ex-9 (50 μg/mouse) 15 minutes prior to i.p. glucose administration. For glucose-stimulated insulin secretion experiments, α-GqD mice and control littermates were fasted overnight and then coinjected i.p. with glucose (2 g/kg) and DCZ (10 μg/kg). Plasma insulin and glucagon levels were measured immediately before and 5, 15, and 30 minutes after injections. Blood was collected from the tail vein.

### ITT.

To assess the effect of activating G_q_ signaling in α cells on insulin sensitivity, α-GqD mice and control littermates were fasted for 4 hours and then injected with human insulin (0.75 U/kg i.p. in saline), in the presence or absence of DCZ (10 μg/kg i.p. in saline), followed by the monitoring of blood glucose levels at regular time intervals. To measure AVP levels during insulin-induced hypoglycemia, WT mice (C57BL/6NTac mice, 8-week-old males) were injected i.p. with insulin (0.75 U/kg) or vehicle (saline). Subsequently, blood samples were taken from the tail vein at regular intervals for blood glucose and plasma AVP measurements. To study the effect of inhibiting endogenous V1bR signaling on insulin tolerance, WT mice (10-week-old males) were fasted 4 hours and then injected i.p. with a selective V1bR antagonist (SSR149415; 25 mg/kg in saline with 5% DMSO and 5% Tween 80) (33) or vehicle. Thirty minutes later, mice received insulin (1.25 U/kg i.p.), followed by the measurement of blood glucose and plasma glucagon levels at defined postinjection time points. Preinjection blood glucose and plasma glucagon levels were also determined. Blood was collected from the tail vein.

### Pyruvate tolerance and glucagon challenge tests.

To assess the effect of chronic activation of α cell G_q_ signaling on hepatic glucose production, HFD α-GqD mice and control littermates were fasted overnight and then injected with sodium pyruvate (1 g/kg i.p.). To measure the ability of glucagon to induce hyperglycemia, HFD α-GqD mice and control littermates that had been fasted for 6 hours received a bolus of glucagon (100 μg/kg i.p.). Blood glucose levels were determined at specific postinjection time points.

### Glucopenia induced by 2-DG.

To measure plasma AVP levels during 2-DG–induced glucopenia, WT mice (C57BL/6NTac mice, 8-week-old males) were fasted for 4 hours and then injected i.p. with 2-DG (500 mg/kg) or vehicle (saline). Blood samples were taken at regular intervals from the tail vein for blood glucose and plasma AVP measurements. A subgroup of WT mice (9-week-old males) were injected i.p. with the V1bR antagonist SSR149415 (25 mg/kg in saline with 5% DMSO and 5% Tween 80) or vehicle 30 minutes prior to 2-DG administration. Plasma glucagon levels were measured as described above.

### Glucagon and insulin secretion studies with isolated mouse islets.

Pancreatic islets were prepared from different mutant mouse strains and WT mice (C57BL/6NTac mice) (mouse age: 16–24 weeks) as described before (58). Islets were cultured overnight in RPMI 1640 medium containing 1% penicillin/streptomycin, 10% FBS, and 7.5 mM glucose. Islets were then placed into perifusion chambers (100 islets/chamber) with Bio-Gel P-4 media (Bio-Rad) in an automated perifusion system (Biorep Perifusion System). The concentrations of the various agents that were added to the perifusion medium are indicated in the main text and in the figure legends. Perifusion experiments were carried out as described previously (59). Glucagon and insulin concentrations in the perfusates were determined with a mouse glucagon ELISA kit (R&D Systems) and a mouse insulin ELISA kit (Mercodia), respectively, following the manufacturers’ instructions.

### Western blotting and immunofluorescence studies.

For Western blotting studies, different mouse tissues (~20 mg) were homogenized in 400 μL of RIPA buffer containing protease and phosphatase inhibitor cocktails. Tissue lysates were centrifuged at 4°C at 14,000*g* for 15 minutes. The supernatants were diluted 20-fold, and protein concentrations were determined using a BCA assay kit (Thermo Fisher Scientific). Subsequently, samples containing 20 mg protein were incubated at 37°C for 10 minutes, and proteins were separated using 3%–8% Tris-acetate SDS-PAGE gels (Thermo Fisher Scientific). Immunoblotting studies were performed using standard procedures. Immunoreactive proteins were visualized by using SuperSignal West Dura Chemiluminescent Substrate (Pierce). The expression of the GqD designer receptor was visualized by using a rabbit anti-HA primary antibody (1:1000; Cell Signaling Technology 3724) (note that the GqD construct contained an N-terminal HA epitope tag), followed by incubation with an HRP-conjugated anti-rabbit secondary antibody (1:5000; Cell Signaling Technology) (for antibody details, see Supplemental Table 1).

To study the expression of the GqD receptor in the mouse pancreatic islet cells, pancreata from α-GqD mice and control littermates were fixed overnight with 4% paraformaldehyde and embedded in paraffin. After deparaffinization, slides were heated in IHC-Tek epitope retrieval solution for 30 minutes and permeabilized in 0.25% Triton X-100 in PBS for 10 minutes. Samples were then blocked in 5% goat serum in PBS and incubated overnight at 4°C with a rabbit anti-HA primary antibody (Cell Signaling Technology 3724), together with an anti-insulin or anti-glucagon antibody (Abcam 7842 and 10988, respectively). After thorough washes with PBS, slides were incubated for 1 hour at room temperature with a mixture of Alexa Fluor 555– or Alexa Fluor 488–conjugated secondary antibodies (1:500 dilution) (for antibody details, see Supplemental Table 1).

To study *V1bR* expression in mouse islets, *Avpr1b-Cre* mice (31) were crossed with a Cre-dependent tdTomato reporter line (Ai9) (The Jackson Laboratory 007909). Doubly transgenic mice resulting from these matings were perfused transcardially with saline, followed by 10% neutral buffered formalin. Mouse pancreata were collected, cryoprotected in 30% sucrose/PBS at 4°C overnight, and then embedded in OCT compound and cryosectioned for immunofluorescence staining. Pancreatic cryosections were blocked with 5% goat serum in PBS and then incubated overnight with anti-insulin or anti-glucagon antibodies as described above. Samples were then extensively washed in PBS, followed by a 1-hour incubation at room temperature with an Alexa Fluor–conjugated secondary antibody (1:500). All sections were counterstained and mounted using ProLong Gold antifade reagent with DAPI. Negative controls were obtained by omitting the primary antibodies. Samples were analyzed by a Keyence digital microscope (BZ-9000) with a CFI Plan Apo λ ×40 lens or by a confocal microscope (Zeiss LSM 700). Detailed information about the antibodies used is given in Supplemental Table 1.

### Quantification of gene expression by reverse transcription quantitative PCR.

For mRNA expression analysis, total hepatic mRNA was extracted and purified using the RNeasy Mini Kit combined with RNase-free DNase set from QIAGEN, according to the manufacturer’s instructions. cDNA was synthesized using SuperScript III First-Strand Synthesis SuperMix (Invitrogen). Reverse transcription quantitative PCR was performed and detected using SYBR Green (Applied Biosystems). mRNA expression data were normalized relative to the expression of β-actin. All PCR primers used are listed in Supplemental Table 1.

### Glucagon release studies with perifused human islets.

Hormone secretion studies were carried out with isolated human islets as described previously (60). Briefly, human islets were received through the Integrated Islet Distribution Program. Islets from 10 different donors were used (for details, see Supplemental Table 2). A total of 800 handpicked islets were placed on a nylon filter in a perifusion chamber (MilliporeSigma) and perfused at a flow rate of 0.8 mL/min. The perifusion apparatus consisted of a programmable, computer-controlled, low-pressure chromatography system (Bio-Rad Econo), a water bath (37°C), and a fraction collector (Waters Fraction Collector III). The perifusion solution was equilibrated with 95% O_2_ and 5% CO_2_ and consisted of Krebs buffer (pH 7.4) containing: 114 mM NaCl, 5 mM KCl, 24 mM NaHCO_3_, 1 mM MgCl_2_, 2.2 mM CaCl_2_, 1 mM Pi, 10 mM HEPES (pH 7.4), and 0.25% BSA.

To assess the effects of a V1bR agonist (d[Leu^4^,Lys^8^]-VP) on glucagon secretion, islets were first pre-perifused with substrate-free medium. Next, a physiological AAM (total concentration: 4 mM) was added, followed by low glucose (G3, 3 mM glucose). In a subset of experiments, a V1bR antagonist (or vehicle) was added, followed by the addition of the V1bR agonist and high glucose (G16.7, 16.7 mM glucose). Last, all compounds were washed out to reestablish basal secretion rates. Each step in this protocol was 20 minutes long (see Figure 6 for the detailed experimental protocol), except that antagonist preincubations lasted for only 10 minutes. The nature and concentrations of the various drugs used are indicated in the main text and in the legend of Figure 6.

Glucagon concentrations in the perifusates were measured by double antibody RIA (GL-32K, MilliporeSigma). Secretion rates were normalized to the final 10 minutes of the G3 step, where a steady-state glucagon secretion rate was established in the presence of AAM and G3.

### Statistics.

Data are expressed as mean ± SEM for the indicated number of observations. Prior to performing specific statistical tests, we performed tests for normality and homogeneity of variance. Data were then tested for statistical significance by mixed effects repeated measures ANOVA for differences after injection, 2-tailed unpaired Student’s *t* test, or 1-way ANOVA, as appropriate. *P* < 0.05 was considered statistically significant.

### Study approval.

All animal studies were approved by the National Institute of Diabetes and Digestive and Kidney Diseases Institutional Animal Care and Use Committee. The University of Pennsylvania Institutional Review Board exempted research in human islets from ethical review because the islets were received from deceased, deidentified organ donors. All pancreata were from deceased donors after having obtained consent from their families through United Network for Organ Sharing.

## Author contributions

LL, DD, NMD, and JW designed and conceived the experiments. LL, DD, KFS, LFB, YC, JCR, and NMD performed and analyzed data from the experiments. YX, JJ, GMK, EK, and KHK provided potentially novel reagents or mouse models. LL and JW wrote the manuscript.

## Supplementary Material

Supplemental data

## Version 1 11/09/2021

In-Press Preview

## Version 2 12/22/2021

Electronic publication
