# Supplementary material for: Gq signaling in α cells is critical for maintaining euglycemia
Source: JCI Insight. 2021 Dec 22;6(24):e152852. doi: 10.1172/jci.insight.152852 (PMC8783673; doi:10.1172/jci.insight.152852)
Supplement: Supplemental data [file jciinsight-6-152852-s030.pdf]

Supplemental Data

α-cell G_q signaling is critical for maintaining euglycemia

Liu Liu, Diptadip Dattaroy, Katherine F. Simpson, Luiz F. Barella, Yinghong Cui, Yan Xiong, Jian Jin, Gabriele M. König, Evi Kostenis, Jeffrey C. Roman, Klaus H. Kaestner, Nicolai M. Doliba, and Jürgen Wess

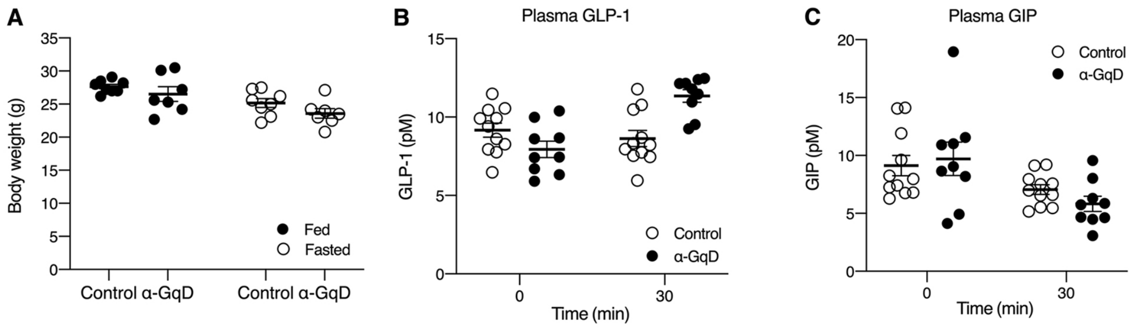

Supplemental Figure 1. Body weight and plasma GLP-1 and GIP levels after DCZ treatment of α-GqD and control mice. (**A**) Body weight of α-GqD mice and control littermates (12-week-old males) that had free access to food or had been fasted overnight for ~14 hr. (**B, C**) Plasma GLP-1 (**B**) and GIP (**C**) levels 30 min after injection of DCZ (10 μg/kg i.p.) (13-week-old male mice with free access to food). Data are given as mean ± SEM (n=7 or 8 for body weight measurements; n=9-11 for hormone measurements).

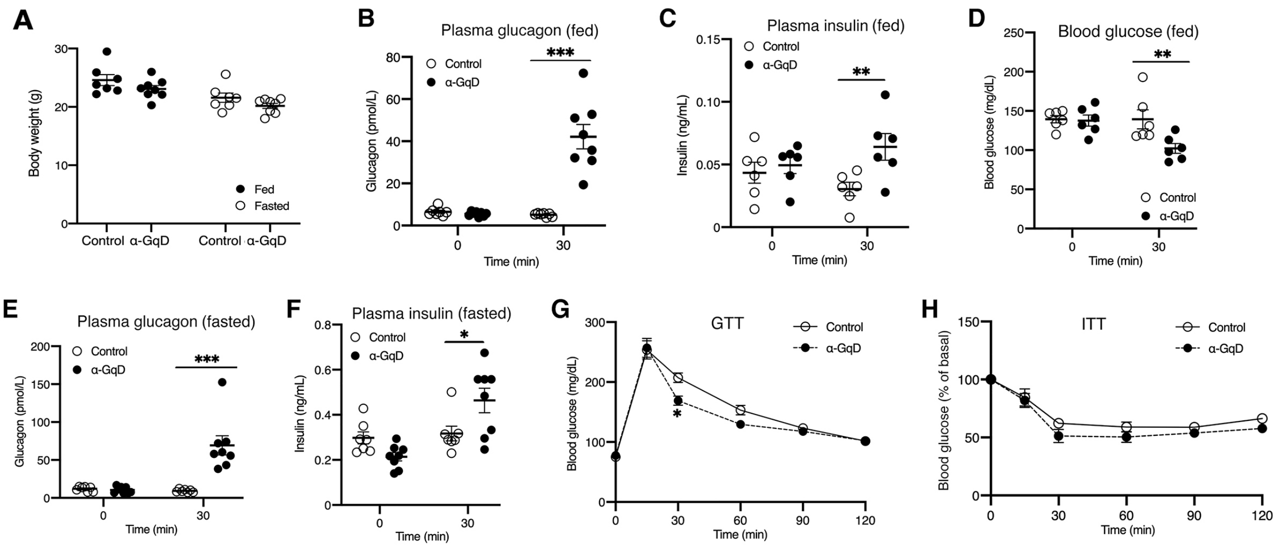

Supplemental Figure 2. Acute activation of α-cell G_q signaling stimulates glucagon and insulin secretion in female mice in vivo. (**A**) Body weight of α-GqD mice and control littermates (age: 15-16 weeks) with free access to food or after an overnight fast (~14 hr). **(B-F)** α-GqD mice and control littermates received a single dose of DCZ (10 µg/kg i.p.), followed by the measurement of plasma glucagon (**B, E**), plasma insulin (**C, F**), and blood glucose (**D**) levels. Plasma glucagon and insulin levels were measured under both fed or fasting (~14 hr overnight fast) conditions. Blood samples were collected from the tail vein at the indicated time points. (**G**) Glucose tolerance test (GTT). Mice (age: 17 weeks) that had been fasted overnight were co-injected i.p. with glucose (2 g/kg) and DCZ (10 µg/kg). (**H**) Insulin tolerance test (ITT). Mice (age: 24 weeks) that had been fasted for 4 hr were co-injected i.p. with insulin (0.75 U/kg) and DCZ (10 µg/kg). All studies were carried out with female mice. Data are given as mean ± SEM (α-GqD: n=7-8; control: n=6-8). *$P \leq 0.05$, **$P \leq 0.01$ and ***$P \leq 0.001$ (one-way ANOVA).

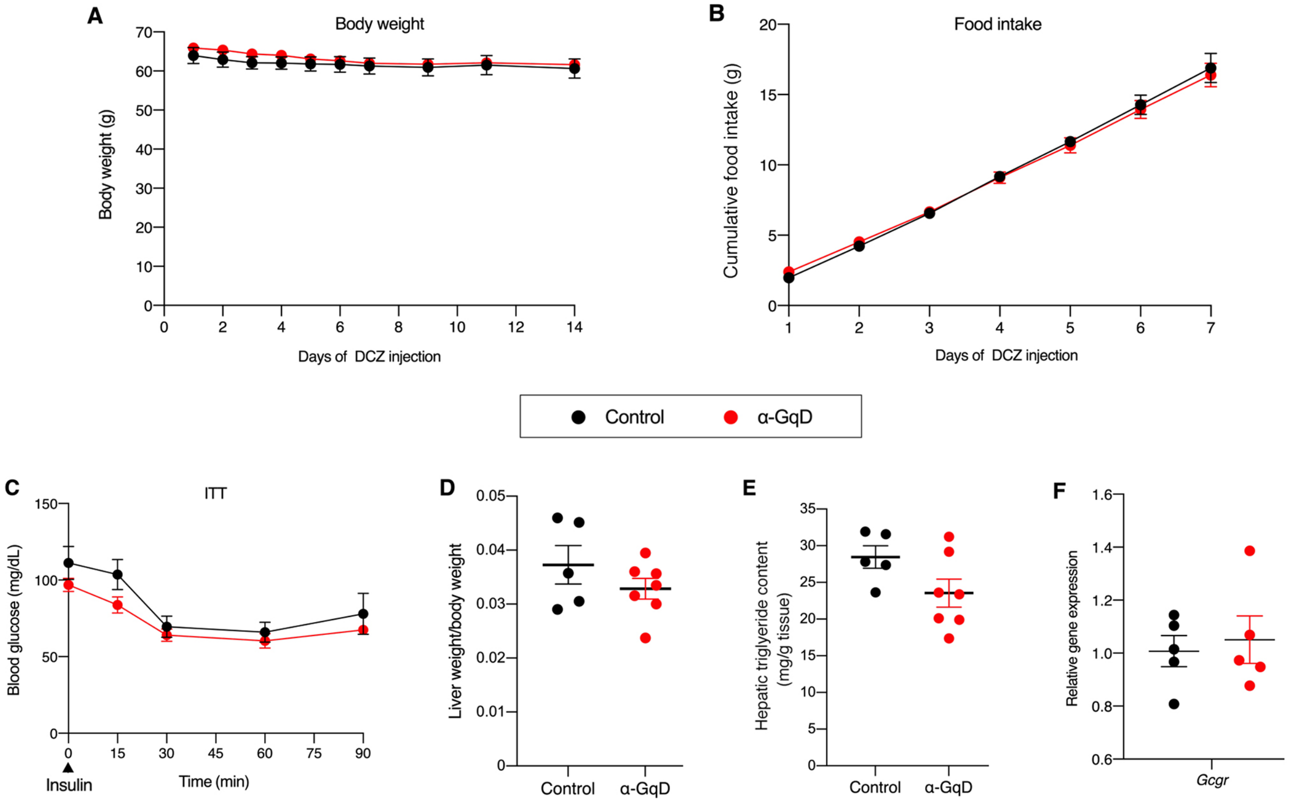

Supplemental Figure 3. Chronic activation of α-cell G_q signaling in mice maintained on a HFD. (**A**) Body weight and (**B**) food intake of α-GqD mice and control littermates maintained on a HFD for 24 weeks. All mice received single daily injections of DCZ (10 µg/kg i.p.). **(C)** Insulin tolerance test (ITT). Mice that had been fasted for 4 hr were injected with insulin (1.25 U/kg i.p.). (**D**) Liver weights (liver weight/total body weight). (**E**) Hepatic triglyceride content. (**F**) Relative hepatic mRNA levels of the mouse glucagon receptor gene (*Gcgr*). The measurements in (**D-F**) were carried out at the end of the DCZ injection period (27 days). Livers were collected after overnight fasting 1 hr after the last DCZ injection. All studies were carried out with male mice (age: ~35 weeks). Data are given as mean ± SEM (n=5-11).

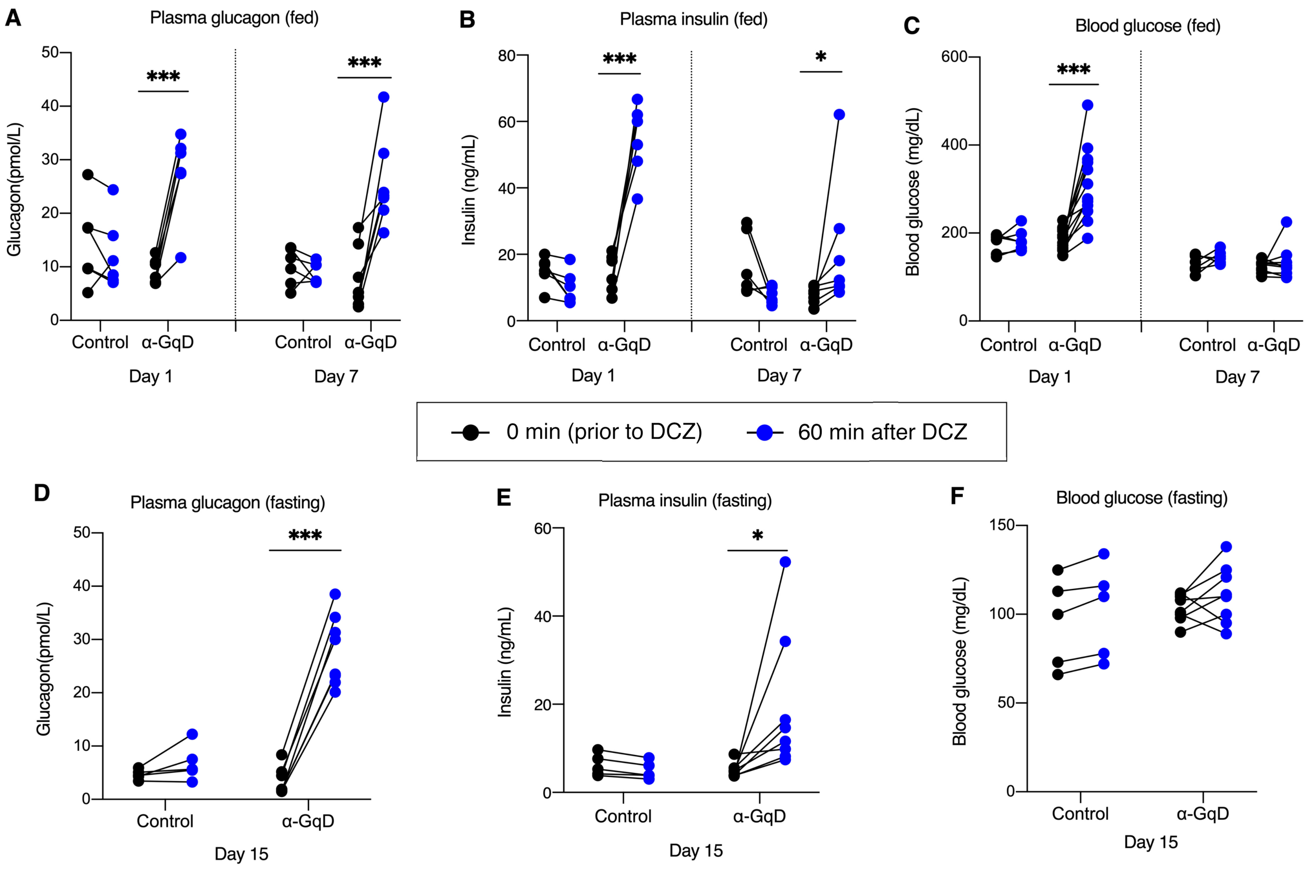

Supplemental Figure 4. Chronic activation of α-cell G_q signaling causes hyperglucagonemia and hyperinsulinemia. (A-F) α-GqD mice and control littermates maintained on a HFD for 24 weeks received daily injections of DCZ (10 µg/kg i.p.). During this time, plasma glucagon (**A, D**), plasma insulin (**B, E**), and blood glucose (**C, F**) levels were monitored. Plasma glucagon, plasma insulin, and blood glucose levels were measured under both fed and fasting (~14 hr overnight fast) conditions 1 hr after DCZ treatment. Data are given as mean ± SEM (n=5-11). *P ≤ 0.05, **P ≤ 0.01 and ***P ≤ 0.001 (one-way ANOVA). All studies were carried out with male mice (age: ~35 weeks).

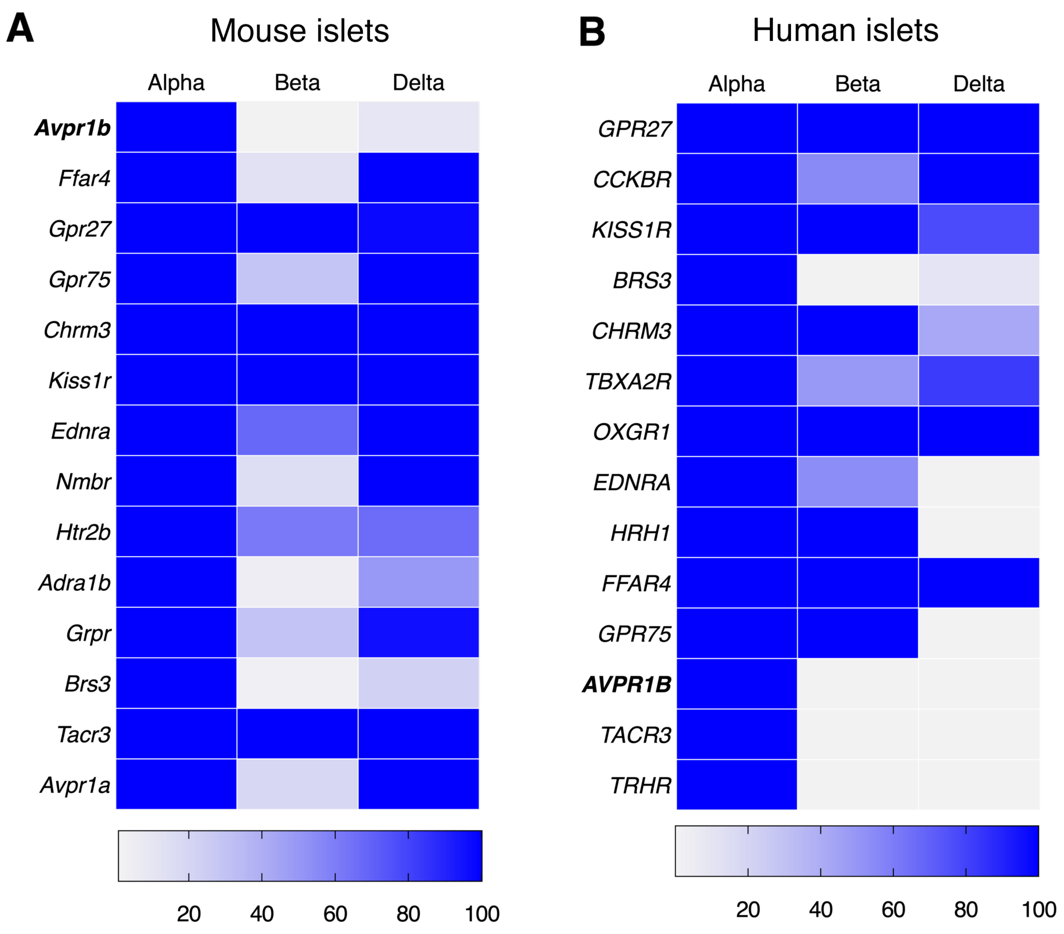

Supplemental Figure 5. Expression profiles of G_q-coupled receptors endogenously expressed by mouse and human islet cells. (A, B) The 14 G_q-coupled receptors with the highest mRNA expression levels in mouse **(A)** and human (**B**) α-cells are listed in descending order of receptor expression. Note that the *Avpr1b*/*AVPR1B* (coding for the V1bR subtype) gene is selectively expressed in both mouse and human α-cells. Data were extracted from published scRNAseq data (1, 2). Receptor expression levels were normalized relative to transcript levels detected in α-cells (=100%).

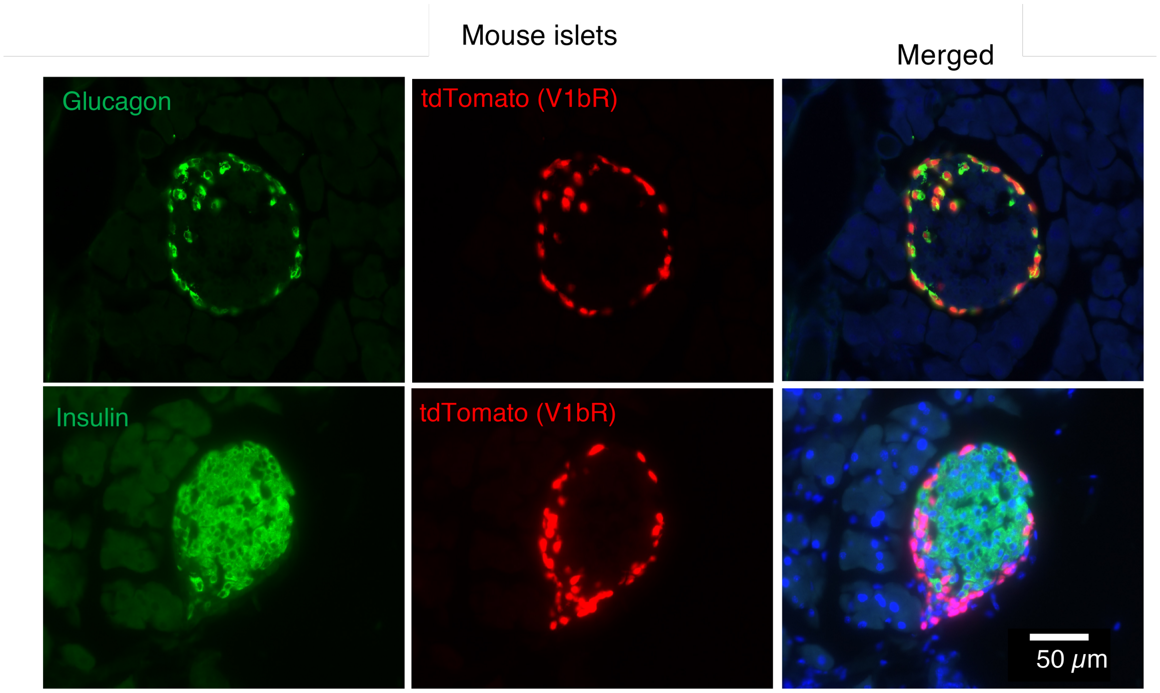

Supplemental Figure 6. Selective expression of the V1bR in mouse pancreatic α-cells. *Avpr1b-Cre* knockin mice were crossed with Cre-dependent tdTomato reporter mice (Ai9) to study *V1bR* expression in pancreatic islets. Pancreatic α-cells were stained with an anti-glucagon antibody (Alexa Fluor, green), and β-cells were visualized with an anti-insulin antibody (Alex Fluor, green), respectively. Nuclei were stained blue with DAPI mounting medium.

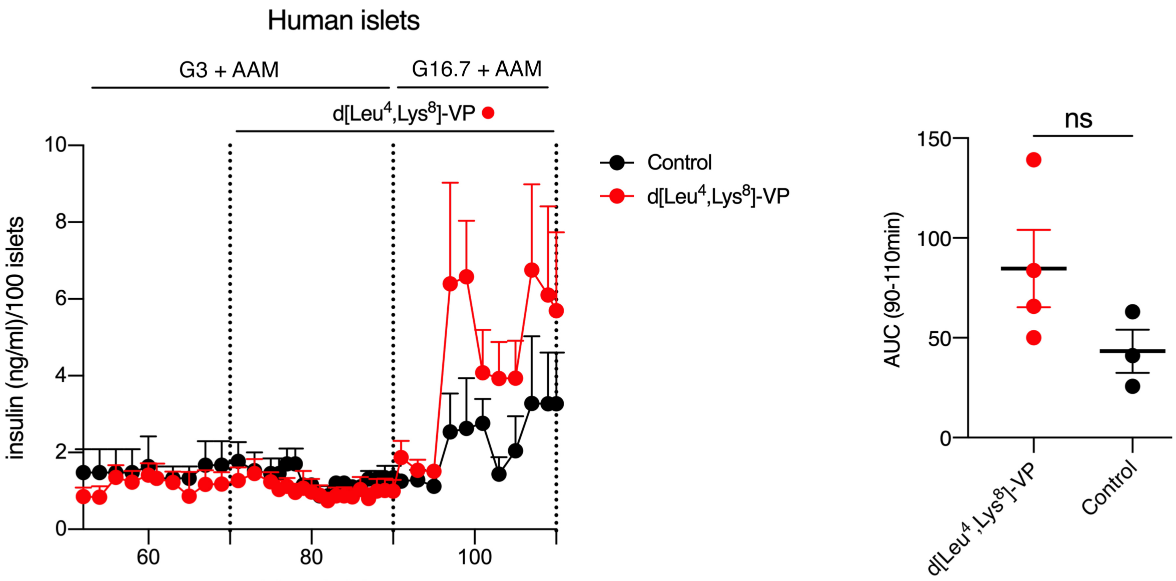

Supplemental Figure 7. Treatment of human islets with a V1bR agonist, followed by insulin secretion measurements. Islets from human donors were perifused with physiological amino acid mixture (AAM) and 3 mM (G3) or 16.7 mM (G16.7) of glucose, respectively, either in the absence (black line) or presence of d[Leu^4, Lys^8]-VP (V1bR agonist; 1 nM, red line). Area under the curve (AUC; arbitrary units) represents insulin secretion during G16.7+AAM. Data are given as mean ± SEM (3-4 perifusions with 800 islets per perifusion chamber per group). ns, no statistically significant difference.

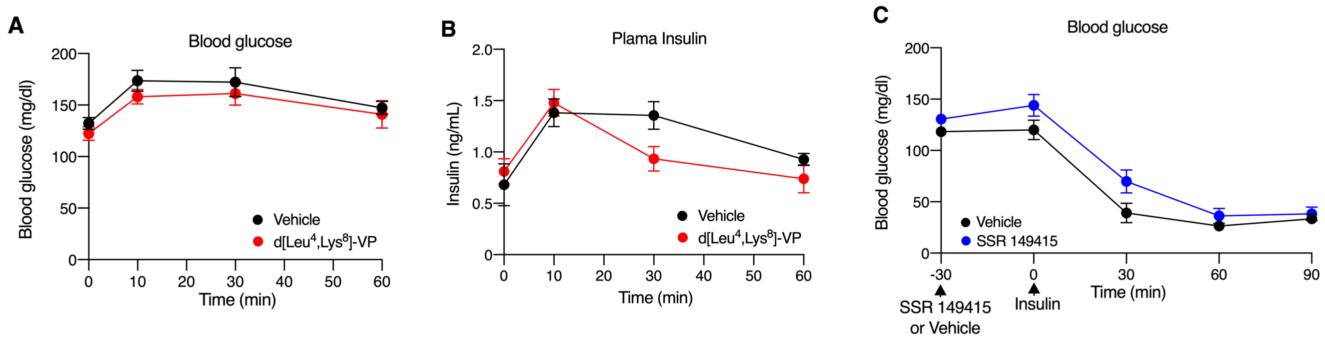

Supplemental Figure 8. Treatment of WT mice with a V1bR agonist or antagonist. (**A, B**) Treatment of WT mice (12-week-old males) with a V1bR agonist. WT mice received a single dose of d[Leu^4, Lys^8]-VP (V1bR agonist; 3 mg/kg, i.v.), followed by the measurement of blood glucose (**A**) and plasma insulin (**B**) levels. (**C**) Lack of effect of V1bR blockade on insulin-induced hypoglycemia. WT mice (10-week-old males) were injected i.p. with either vehicle or SSR149415 (25 mg/kg), a selective V1bR antagonist. Thirty min later, the mice received an i.p. injection of insulin (1.25 U/kg). Data are given as mean ± SEM (n=6 or 7 mice per group).

Supplemental Table 1. Summary of drugs, reagents, PCR primers, and mouse models used

Reagent/resource	Source	Cat #
**Antibodies (Ab)**		
Rabbit anti-HA Ab	Cell Signaling	3724
Guinea pig anti-insulin Ab	Abcam	7842
Mouse anti-glucagon Ab	Abcam	ab10988
Rabbit anti-tdTomato Ab	Clontech	ab632496
Mouse anti-β-actin Ab	Cell Signaling	3700
Anti-rabbit IgG, HRP-linked secondary Ab	Cell Signaling	7074
Anti-mouse IgG, HR-linked secondary Ab	Cell Signaling	7076
Alexa Fluor 555 goat anti-guinea pig secondary Ab	Thermo Fisher Scientific	A-21435
Alexa Fluor 555 goat anti-mouse secondary Ab	Thermo Fisher Scientific	A-28180
Alexa Fluor 488 goat anti-rabbit secondary Ab	Thermo Fisher Scientific	A-11034
Alexa Fluor 488 goat anti-mouse secondary Ab	Thermo Fisher Scientific	A-11017
Alexa Fluor 488 goat anti-guinea pig secondary Ab	Thermo Fisher Scientific	A-11073
**Drugs, reagents, etc.**		
Deschloroclozapine (DCZ)	Dr. Jian Jin (3)	
UBO-QIC	Dr. Evi Kostenis (4)	
d[Leu^4,Lys^8]-VP	Tocris	3127
SSR149415	Tocris	6195
Exendin(9-39) amide	Cayman Chemical	19890
Sodium pyruvate	MilliporeSigma	P2256
Glucagon	Cayman Chemical	24204
2-Deoxy-D-glucose (2-DG)	MilliporeSigma	D8375

Tamoxifen	MilliporeSigma	T5648
Aprotinin	MilliporeSigma	A3428
DPP-4 inhibitor (KR-62436)	MilliporeSigma	K4264
Corn oil	MilliporeSigma	C8267
Human insulin (Humulin R U-100)	Eli Lilly	NDC 0002-8215-17
ProLong^{TM} Gold antifade reagent with DAPI	Thermo Fisher Scientific	P36931
BCA protein assay kit	Thermo Fisher Scientific	23225
ECL Western blotting substrate	Thermo Fisher Scientific	32106
Normal goat serum	Vector Laboratories	S1000
cOmplete, EDTA-free protease inhibitor cocktail	MilliporeSigma	11836170001
PhosSTOP	MilliporeSigma	4906845001
Bovine serum albumin (fatty acid-free)	MilliporeSigma	A7030
Triton X-100	Fisher Scientific	BP151
**Hormone Assay kits**		
Glucagon ELISA kit	Mercodia	10-1281-01
Glucagon RIA kit	MilliporeSigma	GL-32K
Ultra-Sensitive Mouse Insulin ELISA kit	Crystal Chem	9008
Glucagon Quantikine ELISA kit	R&D Systems	DGCG0
Arg^{8}-Vasopressin ELISA kit	Enzo	ADI-900-017A
GLP-1 ELISA kit	Crystal Chem	815
GIP ELISA kit	Crystal Chem	81511
**Mouse strains**		
Gcg-CreER^{T2} mice	Dr. Klaus H. Kaestner (5)	

CAG-LSL-Gq-DREADD mice	Drs. Ute Hochgeschwender and Bryan Roth (6)	Jax # 026220
Avpr1b-Cre knockin mice	Dr. W. Scott Young (7)	
Ai9 dtTomato reporter mice	(8)	Jax #007909
**qRT-PCR primers**		
β-actin (mouse)	Qiagen	QT01136772
Gcgr (mouse)	Eurofins	Forward primer: AGTGACCAATGCCACCACAA Reverse primer: GCCCACACCTCTTGAACACT

Supplemental Table 2. Summary of human islet donors

UNOS/HPAP ID	Recovery Center	Age (Years)	BMI	Sex	Race
RRID:SAMN15724795	Scharp-Lacy	56	32.9	Male	Hispanic
RRID:SAMN15850322	Pennsylvania	52	24.5	Male	Hispanic
RRID:SAMN15877725	Wisconsin	31	27.4	Male	Caucasian
RRID:SAMN16515959	SC-ICRC	51	25.2	Female	Caucasian
RRID:SAMN16734549	Scharp-Lacy	37	28.0	Male	Afr. American
HPAP-074	Pennsylvania	40	36.9	Female	Caucasian
RRID:SAMN17277513	SC-ICRC	43	36.5	Female	Hispanic
RRID:SAMN17528599	Pennsylvania	60	29.9	Male	Caucasian
RRID:SAMN18092805	SC-ICRC	56	21.6	Male	Asian
RRID:SAMN18196260	SC-ICRC	41	28.0	Female	Afr. American

References

1. Xin Y, Kim J, Okamoto H, Ni M, Wei Y, Adler C, et al. RNA Sequencing of single human islet cells reveals type 2 diabetes genes. *Cell Metab.* 2016;24(4):608-15.
2. DiGruccio MR, Mawla AM, Donaldson CJ, Noguchi GM, Vaughan J, Cowing-Zitron C, et al. Comprehensive alpha, beta and delta cell transcriptomes reveal that ghrelin selectively activates delta cells and promotes somatostatin release from pancreatic islets. *Molecular metabolism.* 2016;5(7):449-58.
3. Nagai Y, Miyakawa N, Takuwa H, Hori Y, Oyama K, Ji B, et al. Deschloroclozapine, a potent and selective chemogenetic actuator enables rapid neuronal and behavioral modulations in mice and monkeys. *Nature neuroscience.* 2020;23(9):1157-67.
4. Schrage R, Schmitz AL, Gaffal E, Annala S, Kehraus S, Wenzel D, et al. The experimental power of FR900359 to study Gq-regulated biological processes. *Nat Commun.* 2015;6:10156.
5. Ackermann AM, Zhang J, Heller A, Briker A, and Kaestner KH. High-fidelity Glucagon-CreER mouse line generated by CRISPR-Cas9 assisted gene targeting. *Molecular metabolism.* 2017;6(3):236-44.
6. Zhu H, Aryal DK, Olsen RH, Urban DJ, Swearingen A, Forbes S, et al. Cre-dependent DREADD (Designer Receptors Exclusively Activated by Designer Drugs) mice. *Genesis (New York, NY : 2000).* 2016;54(8):439-46.
7. Williams Avram SK, Lee HJ, Fastman J, Cymerblit-Sabba A, Smith A, Vincent M, et al. NMDA Receptor in Vasopressin 1b Neurons Is Not Required for Short-Term Social Memory, Object Memory or Aggression. *Front Behav Neurosci.* 2019;13:218.
8. Madisen L, Zwingman TA, Sunkin SM, Oh SW, Zariwala HA, Gu H, et al. A robust and high-throughput Cre reporting and characterization system for the whole mouse brain. *Nature neuroscience.* 2010;13(1):133-40.
